# Hip biomechanics in patients with low back pain, what do we know? A systematic review

**DOI:** 10.1186/s12891-024-07463-5

**Published:** 2024-05-28

**Authors:** Gustavo Zanotti Pizol, Gisela Cristiane Miyamoto, Cristina Maria Nunes Cabral

**Affiliations:** Master’s and Doctoral Program in Physical Therapy, Universidade Cidade de São Paulo, Rua Cesário Galeno 475, Tatuapé, Sao Paulo, SP CEP: 03071-000 Brazil

**Keywords:** Hip, Low back pain, Biomechanics, Range of motion, Strength, Electromyography, Kinematic, Systematic review

## Abstract

**Background:**

Biomechanical alterations in patients with low back pain (LBP), as reduced range of motion or strength, do not appear to be exclusively related to the trunk. Thus, studies have investigated biomechanical changes in the hip, due to the proximity of this joint to the low back region. However, the relationship between hip biomechanical changes in patients with LBP is still controversial and needs to be summarized. Therefore, the aim of this study was to systematically review observational studies that used biomechanical assessments in patients with non-specific LBP.

**Methods:**

The search for observational studies that evaluated hip biomechanical variables (i.e., range of motion, kinematic, strength, and electromyography) in adults with non-specific acute, subacute, and chronic LBP was performed in the PubMed, Embase, Cinahl and Sportdiscus databases on February 22^nd^, 2024. Four blocks of descriptors were used: 1) type of study, 2) LBP, 3) hip and 4) biomechanical assessment. Two independent assessors selected eligible studies and extracted the following data: author, year of publication, country, study objective, participant characteristics, outcomes, and results. The methodological quality of the studies was assessed using the Epidemiological Appraisal Instrument and classified as low, moderate, and high. Due to the heterogeneity of the biomechanical assessment and, consequently, of the results among eligible studies, a descriptive analysis was performed.

**Results:**

The search strategy returned 338 articles of which 54 were included: nine articles evaluating range of motion, 16 evaluating kinematic, four strength, seven electromyography and 18 evaluating more than one outcome. The studies presented moderate and high methodological quality. Patients with LBP, regardless of symptoms, showed a significant reduction in hip range of motion, especially hip internal rotation, reduction in the time to perform functional activities such as sit-to-stance-to-sit, sit-to-stand or walking, greater activation of the hamstrings and gluteus maximus muscles and weakness of the hip abductor and extensor muscles during specific tests and functional activities compared to healthy individuals.

**Conclusion:**

Patients with LBP present changes in range of motion, task execution, activation, and hip muscle strength when compared to healthy individuals. Therefore, clinicians must pay greater attention to the assessment and management of the hip during the treatment of these patients.

**Systematic review registration:**

International Prospective Register of Systematic Reviews (PROSPERO) (CRD42020213599).

**Supplementary Information:**

The online version contains supplementary material available at 10.1186/s12891-024-07463-5.

## Background

Low back pain (LBP) is characterized as pain from below the last ribs to the gluteal margin [1–4]. It can be classified according to the duration of symptoms as acute LBP, lasting up to six weeks, subacute, from six to 12 weeks, or chronic, lasting more than 12 weeks [2]. More than 85% of cases do not have a specific cause and, therefore, are diagnosed as non-specific LBP of musculoskeletal origin [3]. LBP has affected millions of people over the years, both in high-income countries and in middle- and low-income countries [5, 6]. Treatment recommendations for LBP vary depending on their classification [1, 3, 4, 7, 8]. Physical activity, manual therapy, and supervised exercise are recommendations for patients with acute and subacute LBP [1, 4, 8, 9]. For the treatment of chronic non-specific LBP, the guidelines recommend exercise as the first line of treatment, without specifying the best type of exercise[1–4, 9].

Studies have already been conducted to understand the biomechanical behavior and the changes in the body regions involved during episodes of LBP [10–13]. Biomechanical changes in patients with LBP are not exclusively related to the trunk. The proximity of the hip joint to the lumbar region directed biomechanical investigations to the hip [14–19], and several studies on the topic were carried out. These studies investigated the association between hip range of motion and non-specific LBP[19], compared the lower limb muscle strength of patients with LBP to the strength of healthy individuals [15], performed kinematic analysis of the hip during sitting and lifting movements in patients with LBP [14], and investigated the activation of hip muscles in patients with LBP during the standing position [16], movements in the sagittal plane[18]and in the prone hip test [17]. These biomechanical studies[14–19] showed different results, therefore a summary of their findings would facilitate the understanding of the role of the hip joint in non-specific LBP. Thus, the present study aimed to systematically review observational studies that used biomechanical hip assessment in patients with non-specific LBP.

## Methods

### Study design

Systematic review written according to the guidelines of the Preferred Report Items for Systematic Review and Meta-Analyses (PRISMA) and prospectively registered in the International Prospective Register of Systematic Reviews (CRD42020213599).

### Inclusion criteria

We included observational studies, i.e., cross-sectional, cohort, and case–control studies, that performed biomechanical hip assessment in patients with acute, subacute, and chronic non-specific LBP, by measuring muscle strength, range of motion, kinematics, muscle activation, balance, or posture. Patients of both sexes should be over 18 years of age. Studies with pregnant women and patients diagnosed with LBP due to nerve root compromise and severe causes, such as neoplasms, inflammatory diseases, infections, and traumas [3] were not included. In studies that presented data from patients with non-specific LBP and another diagnosis, only data from patients with non-specific LBP were extracted. Eligible studies had to be published in full in peer-reviewed scientific journals.

### Search strategy

The search was performed in the following databases: PubMed, Embase, Cinahl, and Sportdiscus on February 22nd, 2024. The descriptors used were extracted from the Medical Subject Heading (MeSH) and divided into four blocks: 1) type of study, 2) LBP, 3) hip, and 4) biomechanical assessment (Additional file 1). The descriptors were combined to perform the searches with OR between the terms of each block and AND between the blocks. There was no restriction on language and date of publication.

### Study selection

Two independent reviewers (GZP and CMNC) conducted the selection process of the studies, first considering the title and abstract, and then the full reading of the study. Disagreements between reviewers were resolved initially by discussion and, in case of persistence, a third reviewer (GCM) reached a consensus.

### Data extraction

Data were extracted by two independent reviewers (GZP and GCM) using a customized spreadsheet. The spreadsheet contained bibliometric data such as date of publication of the study, country, language, and authors; objectives of the study; personal and clinical characteristics of the patients such as age, sex, and duration of pain; sample size; description of the type of study; biomechanical variables and description of the assessment; as well as results. Disagreements between reviewers were resolved initially by discussion and, in case of persistence, a third reviewer (CMNC) reached a consensus.

### Methodological quality assessment

The methodological quality of the included studies was assessed using the Epidemiological Appraisal Instrument [20]. This instrument consists of 43 questions divided into five scales: 1) description, with 17 questions; 2) subject selection, with seven questions; 3) measurement quality, with 10 questions; 4) data analysis, with seven questions; and 5) generalization of results, with two questions. Each question was scored on a scale of 0 to 2, where 0 is “no” or “not informed”, 1 is “maybe” or “partial”, and 2 is “yes”. Questions not applicable to the type of study were disregarded. The scale score was calculated by adding the scores of each question and dividing the total by the number of questions applicable to the type of study used in the assessment. Case–control studies were evaluated with 38 questions, cohort studies with 39 questions, and cross-sectional studies with 34 questions. The methodological quality of the studies was classified as low when the studies had scores between 0 and 0.65, moderate when the studies had scores between 0.7 and 1.35, and high when the studies had scores between 1.4 and 2 [21]. The scale score and the methodological quality classification were performed by two independent reviewers (GZP and GCM). In case of disagreement, a third reviewer (CMNC) reached a consensus.

### Data analysis

The results extracted from the studies were presented descriptively: mean and standard deviation per group for the case–control and cross-sectional studies and by effect size and confidence interval for the cohort studies. When these data were not presented in the study, two emails with the request were sent to the authors within seven days. If there was no response, the data were presented as provided in the study. The results were grouped by type of biomechanical assessment (range of motion, strength, kinematics, and electromyography) and, later, by type of observational study (cross-sectional, case–control, and cohort) and classification of LBP (acute, subacute, and chronic). In studies with more than one type of biomechanical assessment, the outcomes were presented separately in the text considering the biomechanical assessment (some studies were cited more than once throughout the text). Due to the heterogeneity found in the biomechanical hip assessments in patients with LBP, it was not possible to group the results into meta-analyses.

## Results

### Study selection

**Fig. 1:**
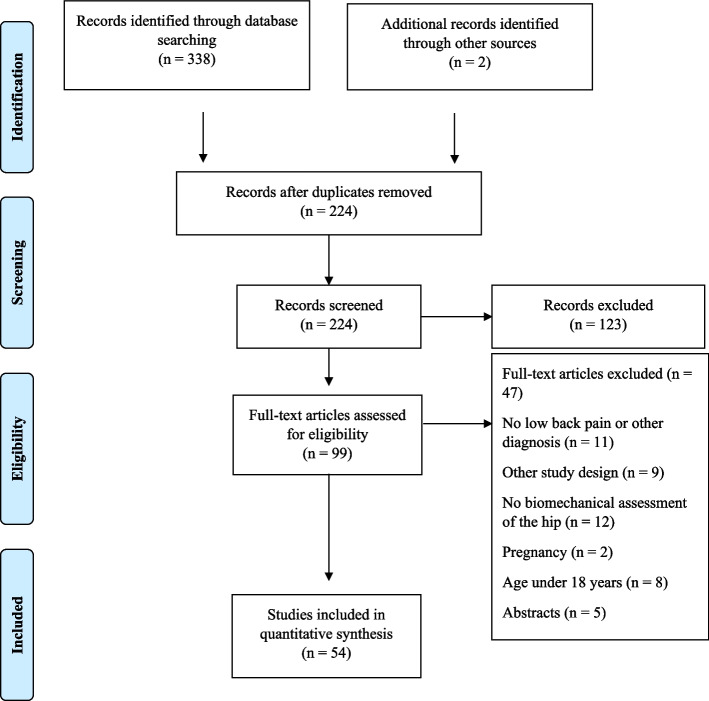
Preferred Reporting Items for Systematic Reviews and Meta-Analyses guidelines (PRISMA) flow diagram summarizing study selection processes

The search strategy returned 338 studies: 116 duplicates were excluded and 123 were excluded after reading the titles and abstracts. Of the 99 studies for full-text reading, 47 were excluded: 11 because the participants did not present LBP, nine because they were not observational studies, 12 because they did not perform biomechanical hip assessment, two because they included pregnant women, eight because they included participants under 18 years of age, and five because they were abstracts presented at conferences. The authors of the abstracts were contacted to clarify whether the studies were published in a scientific journal. As a result, one study was added after contact with the authors. Another study was added after manual search. In total, 54 studies were included for data extraction (Fig. 1).

### Methodological quality

None of the studies presented poor methodological quality (Table 1). Of the studies on range of motion, eight [22–29] presented moderate methodological quality and one [30] had high methodological quality. Three studies on strength [31–33] had moderate methodological quality and one [34] had high methodological quality. Of the studies on kinematics, 13 [35–47] presented moderate methodological quality, and three [48–50] had high methodological quality. Of the studies on electromyography, five [17, 18, 27, 51, 52] presented moderate methodological quality, and two [53, 54] had high methodological quality. Finally, of the studies that evaluated more than one outcome, 14 [16, 55–67] presented moderate methodological quality, and four [68–71] presented high methodological quality.

**Table 1:** Methodological quality of the included studies

**Range of motion**				
**Study**	**Design**	**LBP classification**	**Methodological quality**	
			**Points (0 – 2)**	**Classification**
Nagai et al., 2015 [25] United States	Cross-sectional	Acute	1.15	Moderate
Prather et al., 2017 [26] United States	Cross-sectional	Subacute and chronic	1.24	Moderate
Becker et al., 2022 [72] Germany	Cross-sectional	Chronic	1.11	Moderate
Murray et al., 2009 [24] United Kingdom	Case–control	Chronic	1.15	Moderate
Roach et al., 2015 [29] United States	Case–control	Chronic	1.09	Moderate
Van Dillen et al., 2008 [28] United States	Case–control	Chronic	1.09	Moderate
Cejudo et al., 2020 [22] Spain	Cohort study	Chronic	1.29	Moderate
Handrakis et al., 2012 [23] United States	Cross-sectional	NR	1.15	Moderate
Tak et al., 2020 [30] Netherlands	Cross-sectional	NR	1.62	High
**Strength**				
Arab et al., 2010 [33] Iran	Cross-sectional	Acute, subacute and chronic	1.21	Moderate
Arab et al., 2019 [31] Iran	Cross-sectional	Acute, subacute and chronic	1.18	Moderate
Cooper et al., 2015 [32] United States	Cross-sectional	Chronic	1.24	Moderate
Pizol et al., 2023 [34] Brazil	Cross-sectional	Chronic	1.43	High
**Kinematic**				
Shum et al., 2005 [49] Hong Kong	Cross-sectional	Subacute	1.53	High
Asgari et al., 2017 [35] Iran	Cross-sectional	Chronic	0.94	Moderate
Galli et al., 2000 [37] Italy	Cross-sectional	Chronic	0.97	Moderate
Jimenes del Bairro et al., 2020 [48] Spain	Cross-sectional	Chronic	1.50	High
Scholtes et al., 2009 [42] United States	Cross-sectional	Chronic	1.21	Moderate
Sung, 2013 [44] South Korea	Cross-sectional	Chronic	1.09	Moderate
Ippersiel et al., 2021 [47] Canada	Case–control	Subacute and chronic	0.94	Moderate
Schelldorfer et al., 2015 [50] Switzerland	Case–control	Subacute and chronic	1.41	High
Hart et al., 2009 [38] United States	Case–control	Chronic	1.06	Moderate
Mok et al., 2007 [39] Australia	Case–control	Chronic	0.82	Moderate
Rudy et al., 2003 [40] United States	Case–control	Chronic	0.91	Moderate
Sanchez Zuriaga et al., 2015 [41] Spain	Case–control	Chronic	1.18	Moderate
Scholtes et al., 2010 [43] United States	Case–control	Chronic	1.24	Moderate
Thomas & France, 2007 [45] United States	Cohort	Subacute	1.00	Moderate
Claeys et al., 2012 [36] Belgium	Cross-sectional	NR	1.09	Moderate
Wei et al., 2023 [46] China	Case–control	NR	1.03	Moderate
**Electromyography**				
Kim et al., 2014 [17] South Korea	Cross-sectional	Chronic	1.26	Moderate
Larivière et al., 2010 [53] Canada	Cross-sectional	Chronic	1.43	High
Leinonem et al., 2000 [18] Finland	Cross-sectional	Chronic	1.06	Moderate
Psycharakis et al., 2019 [27] Scotland	Cross-sectional	Chronic	1.29	Moderate
Oliveira et al., 2017 [51] Brazil	Case–control	Chronic	0.94	Moderate
Orakifar et al., 2018 [52] Iran	Case–control	Chronic	1.18	Moderate
Othman et al., 2023 [54] Malaysia	Case–control	NR	1.41	High
**Combined**				
Halbertsma et al., 2001 [58] Netherlands	Cross-sectional	Subacute and chronic	0.82	Moderate
Alsufiany et al., 2020 [70] United States	Cross-sectional	Chronic	1.48	High
Jhonson & Thomas, 2010 [60] United States	Cross-sectional	Chronic	0.88	Moderate
Ringheim et al., 2015 [62] Norway	Cross-sectional	Chronic	1.00	Moderate
Shojaei et al., 2017 [63] United States	Case–control	Acute	1.09	Moderate
Adhia et al., 2016 [68] New Zealand	Case–control	Chronic	1.53	High
Choi et al., 2022 [66] South Korea	Case–control	Chronic	1.23	Moderate
Hamill et al., 2009 [59] United States	Case–control	Chronic	0.91	Moderate
Park et al., 2012 [69] South Korea	Case–control	Chronic	1.40	High
Vatandoost et al., 2022 [67] Iran	Case–control	Chronic	1.31	Moderate
Vogt et al., 2003 [65] Germany	Case–control	Chronic	1.09	Moderate
Busssey et al., 2016 [16] New Zealand	Cohort	Acute and subacute	1.10	Moderate
Crosbie et al., 2013 [56] Australia	Cohort	Chronic	0.97	Moderate
Haddas et al., 2019 [57] United States	Cohort	Chronic	0.92	Moderate
Hicks et al., 2021 [71] United States	Cohort	Chronic	1.50	High
Van Wingerden et al., 2008 [64] Netherlands	Cohort	Chronic	1.09	Moderate
Ciesielska et al., 2015 [55] Poland	Cross-sectional	NR	0.83	Moderate
Jones et al., 2012 [61] United States	Cross-sectional	NR	0.82	Moderate

*LBP* Low back pain, *NR* not reported

### Characteristics of the included studies

Of the 54 studies included, nine assessed range of motion [22–26, 28–30, 72], 16 assessed kinematics [35–43, 45–50, 68], four strength [31–34], seven electromyography [17, 18, 27, 51–54], and 18 assessed more than one outcome (six assessed kinematics and range of motion [44, 56, 59, 60, 63, 69], three range of motion and electromyography [58, 65, 66], two strength and range of motion [67, 71], one strength, range of motion, and electromyography [16], one electromyography and kinematics [55], two electromyography and kinematics [57, 61], one electromyography, kinematics, and strength [62] and two kinematics and strength[64, 70].

### Results of the included studies

Nine inquiries regarding missing descriptive data were sent via email to the authors. Only one study author responded that the requested data were no longer available. Thus, no additional data provided by the authors were included in this systematic review. The objectives of the included studies, characteristics of the sample, outcomes assessed, and the main results are shown in Table 2.

**Table 2:** Results of the included studies

Range of motion				
**Study**	Aim	Participants’ characteristics^a^	Outcomes	Result^a^
**Nagai et al., 2015 **[25] **United States**	To compare hip ROM characteristics of helicopter pilots with and without a self-reported history of LBP	LBP *n* = 30, without LBP *n* = 30 Age LBP: 31.6 ± 5.9 years, without LBP: 31.6 ± 6.0 years Pain: 5.3 ± 2.2, measured by the Numerical pain rating scale (0–10) Disability: 18.3 ± 16.6, measured by the Oswestry disability index (0–100)	Hip internal and external rotation ROM assessed using an inclinometer	LBP group had significantly greater side-to-side asymmetry in the total hip rotation ROM (LBP: 0.95 ± 0.03º, without LBP: 0.97 ± 0.04º, *p* = 0.037)
**Prather et al., 2017 **[26] **United States**	To prospectively collect observational cross-sectional data regarding hip physical examination findings in patients presenting for evaluation and treatment of LBP	Total *n* = 101 Age: 47.6 ± 14 years Sex: 67% female Pain: average 4 (3, 6), worst 9 (7, 10), least 2 (0, 4), measured by the Numerical pain rating scale (0–10) (values presented as median (interquartile range [25th, 75th percentile]) Disability: 34.5 ± 18, measured by the modified Oswestry disability index (0–100), 11.4 ± 6, measured by the Roland Morris disability questionnaire (0–24)	Hip flexion, hip internal rotation with the hip flexed to 90°, and hip external rotation with the hip flexed to 90° ROM assessed using a goniometer	Mean hip flexion was significantly smaller for men (97.3 ± 5º) than for women (102 ± 7º) (*p* < 0.001) Mean hip internal rotation with the hip flexed to 90° was also significantly smaller for men (12.5 ± 8º) than for women (19.1 ± 7º) (*p* < 0.001) Significantly more men (30%) than women (10%) had normal hip flexion ROM and hip internal rotation with the hip flexed to 90° (*p* = 0.008)
**Becker et al., 2022** [72] **Germany**	To assess the validity of the finger-floor distance as a parameter to represent hip mobility in participants with LBP over those without	LBP *n* = 167, without LBP *n* = 365 Age LBP: male 49 (22) years, female 51 (20) years; without LBP: male 36 (21) years, female 37 (22) years [median (interquartile)] Sex: LBP 70 male, 97 female; without LBP 159 male, 197 female	Finger-floor distance and pelvic ROM assessed using an Epionics-SPINE (a three-dimensional accelerometer)	LBP patients had a significantly increase in finger-floor distance compared to the group without LBP (LBP: 16.5 cm (33.3), without LBP: 0.0 cm (15.0), *p* < 0.001) [median (interquartile)] LBP patients had a significantly reduced pelvic version compared to the group without LBP (LBP: 13.5 (11.0), without LBP: 17.7 (11.9), *p* < 0.001) [median (interquartile)]
**Murray et al., 2009** [24] **United Kingdom**	To determine if a significant difference in the lead leg internal hip rotation existed between and within groups of amateur golfers with and without LBP	LBP *n* = 28, without LBP *n* = 36 Age LBP: 56.4 ± 8.4 years, without LBP: 54.3 ± 14.4 years Sex: LBP 2 female, 26 male, without LBP 4 female, 32 male	Internal hip rotation with passive and active ROM for right and left sides assessed using an inclinometer	Between-group comparison: LBP group had a 10º (95% CI 14.6, -5.2) deficit of mean passive internal rotation of the lead hip compared to participants without LBP (*p* < 0.001). The active range deficit was 7º (95% CI 11.1, -2.0, *p* < 0.05) Within-group comparison: LBP group had a 7º (95% CI 11.4, -6) deficit of mean passive internal hip rotation between the lead and non-lead hip (*p* < 0.05)
**Roach et al., 2015** [29] **United States**	To compare passive ROM of hip extension, hip internal rotation and external rotation and total hip rotation in healthy subjects with active subjects with chronic LBP	LBP *n* = 30, without LBP *n* = 30 Age LBP: 45.0 ± 12.0 years, without LBP: 34.0 ± 13.1 years Sex: LBP 16 female, 14 male, without LBP 17 female, 13 male	Internal and external rotation of both left and right hip assessed using a digital inclinometer and hip extension assessed using the modified Thomas test	Hip extension ROM was significantly greater in the group without LBP (6.8 ± 7.2º) than in the chronic LBP group (-4.2 ± 8.8º, *p* < 0.001)
**Van Dillen et al., 2008** [28] **United States**	To examine whether passive hip rotation ROM was different between people with and without LBP who regularly participated in a rotation-related recreational sport	LBP *n* = 24, without LBP *n* = 24 Age LBP: 26.2 ± 7.3 years, without LBP: 27.0 ± 7.7 years Sex: LBP 7 female, 17 male, without LBP 6 female, 18 male Pain: 2.8 ± 1.8, measured by the Verbal numerical pain intensity rating (0–10) Disability: 15.9 ± 8.3, measured by the Oswestry disability index (0–100%)	Passive hip rotation assessed using a fluid filled inclinometer	The LBP group (54.1 ± 2.5º, 95% CI 59, 48.3) had smaller total hip rotation ROM than the group without LBP (61.2 ± 1.78º, 95% CI -63.9, 56.5, *p* < 0.022)
**Cejudo et al., 2020 **[22] **Spain**	To analyze the association between hip ROM and LBP and to establish a diagnostic cutoff for ROM of high risk of LBP	LBP *n* = 14, without LBP *n* = 6 Age: female participants 22.7 ± 3.3 years, male participants 22.3 ± 2.5 years Sex: 10 female, 10 male	Passive hip extension, hip adduction, hip flexion with knee flexed and extended, hip abduction, hip internal rotation, hip external rotation and hip total rotation assessed using an inclinometer	There were statistically significant differences between LBP group and group without LBP in the hip external rotation (62.1 ± 8.2º, 53.2 ± 5.5º, respectively, *p* = 0.013) and hip total rotation (101.3 ± 12º, 89.5 ± 8.7º, respectively, *p* = 0.032) The group of inline hockey players with LBP had an increased range of 8.9º (LBP group 62.1 ± 8.2º, without LBP 53.2 ± 5.5º, *p* = 0.013) and 11.8º (LBP group 101.3 ± 12º, without LBP 89.5 ± 8.7º, *p* = 0.032) in the hip external rotation and hip total rotation, respectively The angles of hip external rotation and hip total rotation that most accurately identified individuals at risk for LBP were 56.5º and 93º, respectively Inline hockey players with hip external rotation ≥ 56.5º and hip total rotation ≥ 93º have 12 times more risk of developing LBP than inline hockey players with hip external rotation < 56.5º and hip total rotation < 93º
**Handrakis et al., 2012 **[23] **United States**	To investigate which of the factors, postural and musculoskeletal impairments, commonly associated with LBP, are different in college-aged persons when grouped by the presence or absence of LBP or the disability resulting from LBP	Total *n* = 84 Age: 24.4 years Sex: 50 female, 34 male Pain: hypolordosis group 56.8, hyperlordosis group 36.6, normal curvature group 44.1, measured by the Visual analog scale (0–100)	Hamstring length assessed using the 90/90 straight leg and hip flexor length using the Thomas test	No association between hamstring length and hip flexor length with pain and disability was found
**Tak et al., 2020 **[30] **Netherlands**	To examine whether male judokas with LBP have lower flexibility of their lumbar spine-hip complex than judokas without LBP	LBP *n* = 29, without LBP *n* = 33 Age LBP: 25.1 ± 5.4 years, without LBP: 24.8 ± 5.2 years Sex: 100% male Pain: 31.4 ± 19.1, measured by the Visual analog scale (0–100)	Hip ROM using a digital inclinometer and Fingertip to floor distance	For the non-dominant hip both passive and active total and internal rotation ROM were significantly lower in judokas with LBP than in those without (*p* < 0.001) Active internal rotation of the dominant hip was lower in the LBP group (28.4 ± 7.9º) than in the group without LBP (33.5 ± 4º, *p* = 0.002)
**Strength**				
**Arab et al., 2010 **[33] **Iran**	To evaluate the relationship between tightness of iliotibial band and hip abductor muscle strength in subjects with LBP	Total *n* = 300 Age LBP with shortened iliotibial band: 44.2 ± 13.0 years, LBP without shortened iliotibial band: 42.6 ± 14.0 years, without LBP: 43.4 ± 4.4 years	Strength of hip abductor muscle assessed using a pressure meter	Subjects without LBP had significantly greater hip abductor muscle strength (33.5 ± 7.3 kPa) compared to subjects with LBP with shortened iliotibial band (27.1 ± 8 kPa, *p* < 0.001) and those with LBP without shortened iliotibial band (27.9 ± 8 kPa, *p* < 0.001)
**Arab et al., 2019 **[31] **Iran**	To evaluate whether there is any significant difference in hip extensor strength between participants with and without LBP, and whether these differences could increase the risk of sustaining LBP	LBP *n* = 266, without LBP *n* = 215 Age LBP: female subjects 44.4 ± 12.4 years, male subjects 42.2 ± 14.4 years, without LBP: female subjects 45.4 ± 13.3 years, male subjects 42.2 ± 15.0 years Sex: LBP 127 female, 139 male, without LBP 82 female, 133 male	Strength of the hip extensor muscle assessed using a pressure meter	Hip strength was greater in the group without LBP (male 33.8 ± 6.4 kPa, female 23.9 ± 5.4 kPa, *p* < 0.001) compared with patients with LBP (male 27.5 ± 7.5 kPa, female 18.8 ± 5.2 kPa, *p* < 0.001) A weak hip extensor can increase the risk of being classified in the LBP group (Exp (β) 5.2, 95% CI 4.3, 6.5, *p* < 0.001)
**Cooper et al., 2015 **[32] **United States**	To describe the hip abductor weakness in a clinical population with LBP and a matched sample without LBP	LBP *n* = 150, without LBP *n* = 75 Age LBP: 41.4 ± 13.0 years, without LBP: 40.7 ± 13.9 years Sex: LBP 64.7% female, without LBP 63.5% female	Manual muscle tests of the gluteus medius, tensor fasciae latae, and gluteus maximus using break tests, Trendelenburg sign was assessed as a functional measure of gluteus medius strength	In LBP group gluteus medius strength was significantly smaller on the affected side than on the unaffected side or the group without LBP (manual muscular test grade 3.4 ± 0.7, 4.6 ± 0.7, respectively, *p* < 0.001) Tensor fasciae latae strength in the LBP group was significantly greater on the unaffected side than on the group without LBP (manual muscular test grade 5.0 ± 0.3, 4.5 ± 0.5, respectively, *p* < 0.001). Trendelenburg sign in the LBP group was significantly more prevalent on the affected side compared to both the unaffected side and group without LBP (*p* < 0.001)
**Pizol et al., 2023** [34] **Brazil**	To investigate whether adult patients with chronic LBP have changes in strength of the hip abductors, adductors, extensors, and external and internal rotators compared to healthy participants	LBP *n* = 40, without LBP *n* = 40 Age LBP: 32.0 ± 11.3 years, without LBP: 32.0 ± 11.5 years Sex: LBP 26 (65%) female, without LBP 27 (67.5%) female Pain: 6.0 ± 1.6, measured by the Pain numerical rating scale (0–10) Disability: 8.4 ± 3.9, measured by the Roland Morris disability questionnaire (0–24)	Isometric muscle strength of the hip abductor, adductor, extensor, external and internal rotator muscles were assessed bilaterally using a handheld dynamometer	There was a statistically significant difference between groups in the strength values for right hip abductors (LBP 153.5 (43.2), without LBP 181.6 (41), *p* = 0.005), right adductors (LBP 72.7 (30.0), without LBP 91.3 (26.0), *p* = 0.005), right internal rotators (LBP 65.6 (17.1), without LBP 74.2 (15.1), *p* = 0.048), right extensors (LBP 87.6 (36.0), without LBP 108.7 (30.3), *p* = 0.010), left abductors (LBP 144.4 (46.8), without LBP 174.9 (35.7), *p* = 0.002), left adductors (LBP 144.4 (46.8), without LBP 174.9 (35.7), *p* = 0.030), and left extensors (LBP 81.9 (35.1), without LBP 103.4 (32), *p* = 0.007)
**Kinematics**				
**Shum et al., 2005** [49] **Hong Kong**	To investigate the effects of subacute LBP and limitation in straight leg raise on the kinematics of the hip joint during Sit-to-Stand and Stand-to-Sit movement	LBP with a negative straight leg raising test *n* = 30, with a positive straight leg raising test *n* = 30, without LBP *n* = 20 Age LBP with a negative straight leg raising test: 40.9 ± 10.0 years, with a positive straight leg raising test: 38.5 ± 10.2 years, without LBP: 41.7 ± 8.2 years Pain: with a negative straight leg raising test 5.7 ± 1.6, with a positive straight leg raising test 5.9 ± 1.9, measured by the Visual analogue scale (0–10) Disability: with a negative straight leg raising test 10.3 ± 4.5, with a positive straight leg raising test 11.6 ± 4.2, measured by the Roland Morris disability questionnaire (0–24)	Postural alignments, maximum joints ranges, joint velocities, lumbar/hip motion ratios, and relative phase angle during the Sit-to-Stand movement assessed using the 3SPACE Fastrak (the device consists of a source that generates a low-frequency magnetic field that is detected by sensors)	Sit-to-stand: patients with LBP with a negative straight leg raising test (right hip 64 ± 10º, left hip 64 ± 11º) and LBP with a positive straight leg raising test (right hip 66 ± 6º, left hip 67 ± 5º) exhibited significant limitations in peak flexion in both hips when compared with participants without LBP (right hip 89 ± 11º, left hip 87 ± 11º, *p* < 0.05) The velocities of the hips movement during flexion and extension were also found to be significantly decreased for those with LBP (LBP with a negative straight leg raising test during flexion: right hip 47 ± 13ºS^−1^, left hip 45 ± 12ºS^−1^, during extension: right hip 77 ± 29ºS^−1^, left hip 80 ± 27ºS^−1^, LBP with a positive straight leg raising test during flexion: right hip 40 ± 14ºS^−1^, left hip 40 ± 14ºS^−1^, during extension: right hip 64 ± 17ºS^−1^, left hip 68 ± 12ºS^−1^, without LBP during flexion: right hip 69 ± 14ºS^−1^, left hip 68 ± 11ºS^−1^, during extension: right hip 121 ± 25ºS^−1^, left hip 118 ± 23ºS^−1^) when compared with participants without LBP (*p* < 0.05) Stand-to-Sit: Patients with LBP (with a negative straight leg raising test: right hip 66 ± 10º, left hip 66 ± 11º, with a positive straight leg raising test: right hip 64 ± 12º, left hip 63 ± 10º), exhibited significant limitations in hip flexion compared to participants without LBP (right hip 87 ± 11º, left hip 86 ± 10º, *p* < 0.05). The velocities of the hips movement during flexion and extension were also found to be significantly decreased for those with LBP (LBP with a negative straight leg raising test during flexion: right hip 82 ± 19ºS^−1^, left hip 84 ± 19ºS^−1^, during extension: right hip 41 ± 13ºS^−1^, left hip 44 ± 16ºS^−1^, LBP with a positive straight leg raising test during flexion: right hip 69 ± 7ºS^−1^, left hip 72 ± 8ºS^−1^, during extension: right hip 36 ± 9ºS^−1^, left hip 37 ± 10ºS^−1^, without LBP during flexion: right hip 115 ± 21ºS^−1^, left hip 111 ± 10ºS^−1^, during extension: right hip 58 ± 14ºS^−1^, left hip 60 ± 14ºS^−1^) when compared with participants without LBP (*p* < 0.05)
**Asgari et al., 2017** [35] **Iran**	To evaluate the effects of lifting-induced fatigue on the local dynamic stability of the hip in individuals with and without chronic LBP	LBP *n* = 14, without LBP *n* = 14 Age LBP: 25.3 ± 3.5 years, without LBP: 23.2 ± 2.0 years Sex: 100% male	A Vicon motion capture system was used to track the 3D kinematic data of the joints captured during lifting-lowering movements	The results revealed that patients with chronic LBP had lower Lyapunov exponent values than the asymptomatic group; Lyapunov analysis showed that the hip movement about the frontal (*p* < 0.05) and transverse (*p* < 0.01) axes were affected by pain**.** Stability of the hip on all anatomical planes varied significantly by late fatigue, in the sagittal (*p* < 0.05), frontal (*p* < 0.01) and transverse (*p* < 0.05) axes
**Galli et al., 2000 **[37] **Italy**	To compare the analysis of the Sit-to-Stand movement of normal and obese subjects with LBP to evidence the motion strategies typical of these patients	LBP *n* = 30, without LBP *n* = 10 Age LBP: female subjects 39.4 ± 13.7 years, male subjects 41.4 ± 6.0 years, without LBP: female subjects 28.0 ± 3.0 years, male subjects 26.0 ± 3.0 years Sex: LBP 25 female, 5 male, without LBP 5 female, 5 male	3D coordinates of reflective markers and ground reaction forces during the Sit-to-Stand movement assessed using a motion measurement system and a force platform	The first trial of the Sit-to-Stand strategy in patients with LBP was different from that of the control group, 84% of the patients with LBP used a strategy characterized by a limited trunk flexion. This strategy produced a high momentum on knee joint (LBP 0.7 ± 0.12Nm/kg, without LBP 0.3 ± 0.04Nm/kg, *p* < 0.05) and limited the torque on the hip joint (LBP 0.4 ± 0.02Nm/kg, without LBP 0.6 ± 0.1Nm/kg, *p* < 0.05)
**Jimenes del Bairro et al., 2020 **[48] **Spain**	To compare the pelvis and hip kinematics, temporo-spatial variables and extensibility of hip muscles between patients with LBP and an asymptomatic group	LBP *n* = 20, without LBP *n* = 20 Age LBP: 24.6 ± 5.2 years, without LBP: 23.2 ± 5.0 years Sex: LBP 76.7% female, 33.3% male, without LBP 57.1% female, 42.9% male Pain: 5.7 ± 2.2, measured by the Visual analog scale (0–100) Disability: 25.2% ± 8.0, measured by the Oswestry disability index (0–100%)	Kinematics of pelvis and hip joints assessed using 2D software and extensibility of hip muscles assessed using Thomas test and modified Ober test	The LBP group showed decreased muscle extensibility of both legs in the modified Ober test (dominant leg of the LBP group -1.3 ± 5.9º, without LBP 3.7 ± 5.5º, *p* = 0.02; non-dominant leg of the LBP group -7.1 ± 8.2º, without LBP 2.8 ± 7.1º, *p* = 0.001) and of the dominant leg in the Thomas test (LBP group 2.4 ± 7º, without LBP 9 ± 8.2º, *p* = 0.009) The pelvic tilt increased in the LBP group compared to the control group (dominant leg of the LBP group 4.5 ± 2.5º, without LBP 2.4 ± 1.9º, *p* = 0.004; non-dominant leg of the LBP group 7.9 ± 2.7º, without LBP 4 ± 2.3º, *p* < 0.001) The LBP group showed less peak joint hip extension during stance phase compared to the control group (dominant leg of the LBP group 4.6 ± 2.3º, without LBP 6.9 ± 2.1º, *p* = 0.003; non-dominant leg of the LBP group 4.2 ± 1.9º, without LBP 6.8 ± 2º, *p* < 0.001)
**Scholtes et al., 2009 **[42] **United States**	To examine timing of lumbopelvic motion between people with and without LBP during two active lower limb movement tests	LBP *n* = 50, without LBP *n* = 41 Age LBP: 28.2 ± 8.1 years, without LBP: 27.9 ± 7.4 years Sex: LBP 18 female, 32 male, without LBP 19 female, 22 male Pain: 2.9 ± 1.7, measured by the Current pain score (0–10) Disability: 14.6% ± 7.6%, measured by the Oswestry disability index (0–100%)	Limb and lumbopelvic kinematics assessed using a 3D system	Patients with LBP demonstrated a greater lumbopelvic rotation angle (without LBP 4.5 ± 2.6º, LBP 5.8 ± 3º, *p* = 0.02) as well as earlier lumbopelvic rotation during hip lateral rotation (without LBP 0.3 ± 0.3º, LBP 0.2 ± 0.1º, *p* = 0.01) compared to participants without LBP
**Sung, 2013 **[44] **South Korea**	To investigate three-dimensional angular displacement differences of the hips and lumbar spine during squatting activities between subjects with and without chronic LBP	LBP *n* = 15, without LBP *n* = 15 Age LBP: 37.2 ± 14.6 years, without LBP: 41.8 ± 16.8 years Disability: 21.7% ± 7.4%, measured by the Oswestry disability index (0–100%)	Synchronized kinematic data recorded and processed by six digital cameras and a force plate	The LBP group demonstrated decreased lumbar flexion in the sagittal plane (LBP 27.5 ± 1.1º, without LBP 31 ± 1.2º, *p* = 0.03) and increased bilateral hip joint flexion during squatting (right hip, LBP 98.3 ± 2.5º, without LBP 91.6 ± 2º, *p* = 0.03, left hip, LBP 100.8 ± 2.5º, without LBP 94.07 ± 1.8º, *p* = 0.03)
**Ippersiel et al., 2021 **[47] **Canada**	To investigate the extent to which patterns of regional lumbo-pelvic-hip coordination differ between adults with and without LBP during bending. A secondary objective was to explore the relationship between inter-joint coordination between adults with and without LBP	LBP *n* = 16, without LBP *n* = 21 Age LBP: 30 ± 9 years; without LBP 27 ± 10 years Pain: 3.4 ± 1.1, measured by the Numeric pain rating scale (0–10): Disability: 25.3 ± 7.4%, measured by the Oswestry disability index (0–100%)	Joint angles and sagittal joint angles assessed for the left hip during the flexion task using an electromagnetic motion capture system, inter-joint coordination was quantified using Continuous Relative Phase analysis, averaged Continuous Relative Phase curves were determined using the three bending trials across each period, for each participant. From these, the mean absolute relative phase was determined by taking the mean of the ensemble Continuous Relative Phase curve for each joint pair and period to represent the amplitude of the Continuous Relative Phase	The group effect showed that adults with LBP had greater mean absolute relative phase values for inter-joint coordination, indicating more out-of-phase coordination, than the healthy group (mean difference = 24.7º; 95% CI = 3.93, 45.4; Cohen’s d = 0.94 (flexion) and 0.63 (extension), independent of movement period
**Schelldorfer et al., 2015 **[50] **Switzerland**	To examine the sway of the hip and center of pressure during three standing tasks conditions with increasing postural control requirements in patients with and without LBP	LBP *n* = 57, without LBP *n* = 22 Age LBP: 39.2 ± 12.8 years, without LBP: 38.6 ± 11.4 years Sex: LBP 26 female, 31 male, without LBP 14 female, 8 male Disability: 18% ± 6%, measured by the Roland Morris disability questionnaire (0–100%)	Movements of the spine and hip assessed using four inertial measurement units at a sampling frequency of 200 Hz Center of pressure was measured with a Wii-balance board sampling with 200 Hz	Mean absolute deviation position of the LBP group was significantly greater than the group without LBP for the hip and center of pressure in the blind-foam condition, both in the sagittal (hip: 1.7, 95% CI = 1.3, 2.2; center of pressure: 1.3, 95% CI = 1.0, 1.6; *p* < 0.001) and frontal plane (hip: 2.1, 95% CI = 1.6, 2.6, center of pressure: 1.4, 95% CI = 1.1, 1.6, *p* < 0.001) Mean absolute deviation velocity of the LBP group was significantly higher than the group without LBP for the hip and center of pressure in the blind-foam condition, both in the sagittal (hip: 2.0, 95% CI = 1.7, 2.4; center of pressure: 3.8, 95% CI = 3.4, 4.3; *p* = 0.001) and frontal plane (hip: 3.1, 95% CI = 2.6, 3.7, center of pressure: 2.8, 95% CI = 2.5, 3.2, *p* = 0.001), and in the blind-firm condition, both in the sagittal (hip: 0.2, 95% CI = 0.05, 0.3; center of pressure: 0.6, 95% CI = 0.4, 0.7; *p* = 0.001) and frontal plane (hip: 0.2, 95% CI = 0.1, 0.4, center of pressure: 0.4, 95% CI = 0.3, 0.5, *p* = 0.001) Gender significantly affected hip movements in the sagittal plane, with women showing greater mean absolute deviation position (*p* = 0.02) and higher mean absolute deviation velocity (*p* = 0.01)
**Hart et al., 2009** [38] **United States**	To compare 3D trunk and lower extremity joint kinematics during jogging gait before and after lumbar paraspinal fatiguing exercise in people with and without a history of recurrent episodes of LBP	LBP *n* = 25, without LBP *n* = 25 Age LBP: female subjects 22.3 ± 2.7 years, male subjects 22.9 ± 3.5 years, without LBP: female subjects 20.8 ± 1.0 years, male subjects 24.5 ± 4.5 years Sex: LBP 12 female, 13 male, without LBP 12 female, 13 male	Peak joint angles in sagittal plane, frontal plane, and transverse plane angles of the trunk, spine, hip, and knee were calculated with a 10-camera motion analysis system	Patients with recurrent LBP exhibited greater peak hip abduction angles (*p* = 0.03) during jogging gait compared with those without LBP
**Mok et al., 2007 **[39] **Australia**	To evaluate the preparatory movement and resultant displacement of the lumbopelvic region associated with unbalance between movement and stability in people with or without LBP	LBP *n* = 10, without LBP *n* = 10 Age LBP: 29.7 ± 5.3 years, without LBP: 28.5 ± 4.3 years Pain: 2.1 ± 1.9, measured by the Visual analog scale (0–10) Disability: 2.8 ± 1.5, measured by the Roland Morris disability questionnaire (0–24)	Kinematic of the trunk and limbs assessed with an electromagnetic motion analysis system. Data were converted to 3D coordinates using the Motion Monitor software	The resultant flexion of the hip was significantly smaller for the trials with preparatory movement in the LBP group compared with without preparatory movement in the LBP group and without LBP group (*p* < 0.05)
**Rudy et al., 2003 **[40] **United States**	To evaluate how differences in movement patterns changed with repetitive lifting	LBP *n* = 53, without LBP *n* = 53 Age LBP: female subjects 37.6 ± 11.5 years, male subjects 22.9 ± 3.5 years, without LBP: female subjects 35.3 ± 13.2 years, male subjects 35.1 ± 8.1 years Sex: 50% female, 50% male	A motion analysis system used to track infra-red reflecting markers placed on the ankle, apex of the patella, greater trochanter of the femur, and acromion of the shoulder during lifting	Hip flexion at the beginning of the lift was significantly greater for participants without LBP (105.7º vs. 96.0°, *p* < 0.001) Hip flexion angles at the end of the lift were significantly larger for participants without LBP compared with those with LBP (9.3º vs. 3.2°, *p* < 0.001) Participants without LBP had significantly later hip midpoint times at the middle phase (*p* < 0.01) and at the late phase (*p* < 0.001) compared with the hip midpoint times of those with LBP
**Sanchez Zuriaga et al., 2015 **[41] **Spain**	To determine the patterns of lumbopelvic motion during trunk flexion–extension movements and to compare these patterns between patients with recurrent LBP in their pain-free periods and matched asymptomatic subjects	LBP *n* = 15, without LBP *n* = 15 Age LBP: 45.0 ± 11.0 years, without LBP: 41.0 ± 11.0 years Sex: LBP 8 female, 7 male, without LBP 8 female, 7 male	Kinematic analysis of the angular displacement of the lumbar spine and the hip in the sagittal plane performed by means of a 3-dimensional video photogrammetric system	There were no differences between the groups
**Scholtes et al., 2010 **[43] **United States**	To examine how effectively people with and without LBP independently modify lumbopelvic motion during an active limb movement test following standardized, within-session instruction	LBP *n* = 19, without LBP *n* = 20 Age LBP: 27.3 ± 6.6 years, without LBP: 26.5 ± 5.9 years Sex: LBP 9 female, 10 male, without LBP 10 female, 10 male Pain: 1.8 ± 1.0, measured by the Current pain score (0–10) Disability: 13.9% ± 9.2%, measured by the Oswestry disability index (0–100%)	Limbs and lumbopelvic kinematics examined from the start to the end of the hip lateral rotation using a 6-camera motion capture system	Compared to patients with LBP, participants without LBP completed a greater angle of hip lateral rotation prior to the start of the lumbopelvic rotation during the hip lateral rotation test (LBP: 7.7 ± 5.7°, without LBP: 12.5 ± 8.2°, *p* = 0.02)
**Thomas & France, 2007 **[45] **United States**	To examine the motor behavior during the performance of a standardized reaching task in individuals recovering from an episode of LBP	Total *n* = 36 Age related anxiety low group: 24.9 ± 5.8 years, related anxiety high group: 28.8 ± 7.5 years Sex: 36.1% male, 63.9% female	Movements of the trunk and limb segments were assessed using a magnetic based kinematic system that tracks the 3-dimensional coordinates of sensors	For the right hip excursions, there were significant main effects of movement speed (*p* < 0.001), target height (*p* < 0.001), and a significant speed by height interaction (*p* < 0.001). Right hip excursions were greater at fast versus self-paced speed and for lower targets; the effect of movement speed on the hip joint excursions increased as target height decreased
**Claeys et al., 2012 **[36] **Belgium**	To investigate if there is an association between altered proprioceptive postural control and the performance of the Sit-to-Stance-to-Sit movement in patients with LBP and to investigate the role of the pelvis in the performance of the Sit-to-Stance-to-Sit movement	LBP *n* = 106, without LBP *n* = 20 Age LBP: 18.5 ± 0.5 years, without LBP: 18.5 ± 0.5 years Sex: LBP 81 female, 25 male, without LBP 13 female, 7 male Pain: 2.0 ± 2.2 measured by the Numerical pain rating scale (0–10) Disability: 8.8 ± 2.0 measured by the Oswestry disability index (0–100)	Postural sway characteristics assessed using a force plate and trunk and pelvis position changes in space assessed using accelerometers	With the feet on a stable support surface, patients with LBP needed significantly more time to perform the five consecutive Sit-to-Stance-to-Sit movements (LBP: 9.3 ± 1.5 s, without LBP: 8.3 ± 1.2 s, *p* < 0.005) This longer duration was mainly caused by significantly longer stance phase (LBP: 1.2 ± 0.20 s, without LBP: 1.1 ± 0.1 s, *p* < 0.05) With both feet on an unstable support surface, patients with LBP again needed more time compared to those without LBP to perform the five Sit-to-Stance-to-Sit repetitions (LBP: 9 ± 1.2 s, without LBP: 8.3 ± 1.2 s, *p* < 0.005) This longer performance was caused by longer stance phases (LBP: 1.1 ± 0.1 s, without LBP: 1 ± 0.04 s; *p* < 0.05) During the performance of the Sit-to-Stance-to-Sit, there was a significantly delayed anterior pelvic rotation onset in the LBP group (stable 0.1 ± 0.1 s, foam 0.1 ± 0.1 s) compared to those without LBP (stable 0.1 ± 0.08 s, foam –0.05 ± 0.2 s) in both conditions to start up the five Sit-to-Stance-to-Sit repetitions (*p* < 0.005) During the movement sequence, the initiating movement of the pelvis was significantly earlier in the participants without LBP (-0.1 ± 0.1 s) compared to the LBP group (-0.05 ± -0.06 s) when moving from Sit-to-Stance on the foam (*p* < 0.05)
**Wei et al., 2023** [46] **China**	To quantitatively analyze the gait functional characteristics of patients with chronic LBP	LBP *n* = 20, without LBP *n* = 20 Age LBP: 21.4 ± 1.1 years, without LBP: 21.2 ± 1.0 years Sex: LBP 10 female, 10 male, without LBP 10 female, 10 male	Gait spatiotemporal parameters, including walking cycle, step frequency, step length, pace speed, step length difference, percentage of single support phase using a three-dimensional gait analyzer	Compared with the group without LBP, the walking cycle time of the LBP group was prolonged (LBP: 1.1 ± 0.1 s, without LBP: 1.0 ± 0.1 s, *p* = 0.044), the step frequency was reduced (LBP: 114.3 ± 9.0 steps/s, without LBP: 120.0 ± 9.0 steps/s, *p* = 0.043), the step length was shortened (LBP: 120.5 ± 12.7 cm, without LBP: 128.7 ± 12.4 cm, *p* = 0.046), and the walking speed was slower (LBP: 115.1 ± 17.2 cm/s, without LBP: 128.6 ± 14.8 cm/s, *p* = 0.011) When comparing the affected limb of the LBP group with the ipsilateral limb of the group without LBP, the step length was shortened (LBP: 55.8 ± 8.4 cm, without LBP: 63.2 ± 8.9 cm, *p* = 0.010) and the percentage of single support phase was reduced (LBP: 58.2 ± 1.0, without LBP: 58.9 ± 1.0, *p* = 0.041)
**Electromyography**				
**Kim et al., 2014 **[17] **South Korea**	To examine activation patterns of the myofascial chain in women with and without chronic LBP during a prone hip extension task	LBP *n* = 15, without LBP *n* = 15 Age LBP: 44.3 ± 10.8 years, without LBP: 42.9 ± 11.3 years Sex: 100% female Pain: 5.1 ± 1.8, measured by the Visual analog scale (0–10) Disability: 47.0 ± 10.2, measured by the Modified Oswestry disability index (0–100)	EMG signals of the gluteus maximus and hamstring muscle	The EMG amplitudes in ipsilateral gluteus maximus (LBP 29.3 ± 9.6% submaximal voluntary contraction, without LBP 20.6 ± 11% submaximal voluntary contraction, *p* = 0.03) and ipsilateral biceps femoris (LBP 48.3 ± 14.9% maximal voluntary isometric contraction, without LBP 34.5 ± 15.4% maximal voluntary isometric contraction, *p* = 0.02) were significantly greater in women with chronic LBP than in women without chronic LBP during a prone hip extension test
**Larivière et al., 2010 **[53] **Canada**	To examine whether a dynamic back muscle endurance exercise performed in a semi sitting position induces more fatigue in back muscles than in hip extensors in healthy subjects and patients with chronic LBP	LBP *n* = 18, without LBP *n* = 16 Age LBP: 40.0 ± 9.0 years, without LBP: 43.0 ± 8.0 years Sex: 50% female Pain: 3.8 ± 2.3, measured by the Visual analog scale (0–10) Disability: 38% ± 22%, measured by the Roland Morris disability questionnaire (0–100%)	EMG signals of the right and left gluteus maximus and right and left biceps femoris	There were no differences between the groups
**Leinonem et al., 2000 **[18] **Finland**	To compare gluteus maximus and biceps femoris muscle function during sagittal trunk flexion and extension in patients with and without chronic LBP	LBP *n* = 19, without LBP *n* = 19 Age LBP: 45.0 ± 4.3 years, without LBP: 41.6 ± 7.2 years Sex: 100% female Pain: 29.1 ± 20.3, measured by the Visual analog scale (0–100) Disability: 21.3 ± 12.8, measured by the Oswestry disability index (0–50)	Raw surface EMG signals were recorded bilaterally over the gluteus maximus and biceps femoris muscles	The relative activation time of the gluteus maximus was shorter in the patients with LBP (22 ± 7%) than in participants without LBP (28.7 ± 5.7%) during extension (*p* < 0.05)
**Psycharakis et al., 2019 **[27] **Scotland**	To investigate gluteal muscle activation and pain intensity during aquatic and land exercises in people with and without chronic LBP	LBP *n* = 20, without LBP *n* = 20 Age LBP: 33.1 ± 6.3 years, without LBP: 28.5 ± 7.8 years Sex: 100% male Disability: 21.1 ± 11.5%, measured by the Oswestry disability index (0–100%)	EMG measurements of the left and right gluteus maximus and gluteus medius	There were no differences between the groups
**Oliveira et al., 2017 **[51] **Brazil**	To compare the amplitude of the EMG activity of the gluteus maximus during Pilates exercises in women with and without LBP	LBP *n* = 30, without LBP *n* = 30 Age LBP: 35.7 ± 12.9 years, without LBP: 34.2 ± 9.9 years Sex: 100% female	EMG signals of the gluteus maximus muscle	There were no differences between the groups
**Orakifar et al., 2018 **[52] **Iran**	To examine the level of muscle activity, duration of muscle activity and time to peak activation between a control group and two homogeneous LBP subgroups during the sit-to-stand and stand-to-sit functional tasks	LBP lumbar flexion rotation group *n* = 7, LBP lumbar extension rotation group *n* = 15, without LBP *n* = 15 Age LBP lumbar flexion rotation group: 34.4 ± 7.0 years, LBP lumbar extension rotation group: 32.2 ± 5.0 years, without LBP: 32.9 ± 4.5 years Sex: 100% female Pain: LBP lumbar flexion rotation group 1.7 ± 1.1 and LBP lumbar extension rotation group 2.2 ± 1.1, measured by the Visual analog scale (0–10) for current pain, LBP lumbar flexion rotation group 2.4 ± 1.0 and LBP lumbar extension rotation group 2.9 ± 1.1, measured by the Visual analog scale (0–10) for the last week Disability: LBP lumbar flexion rotation group 10.0% ± 4.0% and LBP lumbar extension rotation group 11.2% ± 5.8%, measured by the Oswestry disability index (0–100%)	EMG signals of the right and left medial hamstring and lateral hamstring muscles	Sit-to-stand task: A significant reduced time to peak of the left medial hamstring activation was identified in the lumbar flexion rotation group compared to the group without LBP (lumbar flexion rotation 31.6 ± 14.8 ms, without LBP 45.7 ± 15 ms, *p* = 0.03) Stand-to-sit task: A significant reduced time to peak of the left lateral hamstring activation was found in the lumbar extension rotation group compared to the group without LBP (lumbar extension rotation 48.3 ± 17.9 ms, without LBP 60.4 ± 11.7 ms, *p* = 0.03). A reduced time to peak of the left lateral hamstring activation was also observed in the lumbar flexion rotation group compared to the group without LBP (lumbar flexion rotation 43.9 ± 12.5 ms, without LBP 60.4 ± 11.7 ms, *p* = 0.04)
**Othman et al., 2023 **[54] **Malaysia**	To compare the gluteus maximus and gluteus medius activation in two different functional positions (prone lying and single leg standing), in patients with LBP with and without piriformis syndrome	LBP with piriformis syndrome *n* = 36, LBP without piriformis syndrome *n* = 25, without LBP *n* = 30 Age LBP with piriformis syndrome: 36.7 ± 9.3 years, LBP without piriformis syndrome 34.4 ± 8.2 years, without LBP 28.5 ± 10.0 years Sex: LBP with piriformis syndrome 24 female, 12 male, LBP without piriformis syndrome 17 female, 8 male, without LBP 21 female, 9 male	Muscle activation of the gluteus maximus and gluteus medius using an electromyograph	The LBP group without piriformis syndrome demonstrated the strongest gluteus maximus activation (72.6 ± 27.1 μV, *p* = 0.01) compared to the other two groups (LBP group with piriformis syndrome: 47.4 ± 26.5 μV, without LBP: 59.3 ± 18.5 μV)
**Combined**				
**Halbertsma et al., 2001 **[58] **Netherlands**	To investigate the extensibility, ROM and EMG of the hamstrings in patients with LBP	LBP *n* = 20, Flexible group without LBP *n* = 12, Stiff group without LBP *n* = 8 Age LBP: 33.0 ± 11.0 years, Flexible group without LBP: 28.2 ± 7.6 years, Stiff group without LBP: 29.4 ± 5.6 years Sex: LBP 8 female, 12 male, without LBP 10 female, 10 male	Angular rotations during extensibility using electro goniometers and muscle moments (stiffness) using a calculation of the moment encountered at different joint angles during instrumental straight-leg raising test, which consists of an examination table with a lift-frame, and EMG of the hamstrings	ROM and hamstrings extensibility in the patients with LBP were significantly smaller compared with the Stiff group without LBP (hamstring ROM -10.7 ± 3.6º, Sign t 0.006, pelvic-femoral angle ROM -7.8 ± 3.4º, Sign t 0.027) The onset of the EMG for the patients with LBP was significantly earlier than those of the Stiff group (LBP group 26.8 ± 11.9º, Stiff group 42 ± 8.7º, *p* < 0.05)
**Alsufiany et al., 2020 **[70] **United States**	To examine differences in postural control and strength among subgroups of physically active and inactive participants with and without chronic LBP	LBP *n* = 24, without LBP *n* = 24 Age LBP: 28.8 ± 5.9 years, without LBP: 28.2 ± 4.1 years Sex: 50% female Pain: 3 (3, 7), measured by the Numerical pain rating scale (0–10) (values presented as median [minimum, maximum])	Peak isometric hip flexors, extensors, abductors, and external rotators assessed using a handheld dynamometer Dynamic balance assessed using the Y-Balance test Static balance testing assessed using the Balance Master force platform	For the static balance, there was a significant difference between the inactive patients with LBP (8 ± 4.3ºS^−1^) and inactive healthy participants (2 ± 0.8ºS^−1^, *p* < 0.001) There was no significant group x physical activity interaction effect for strength in all muscles (*p* > 0.5)
**Jhonson & Thomas, 2010 **[60] **United States**	To examine the correlation between hamstring flexibility and hip joint excursions during standardized reaching and forward-bending tasks	Total *n* = 122 Age: recovered 30.9 (19–57 [range]) years, LBP: 22.2 (18–37[range]) years, without LBP: 23.8 (18–37 [range]) years Sex: recovered 44 female, 42 male, LBP 10 female, 8 male, without LBP 10 female, 8 female	Hamstring flexibility assessed with the knee straight using a bubble goniometer Hip flexion ROM assessed with the knee bent using a bubble goniometer Joint motions assessed using a Motion Monitor, a magnetic based kinematic system	The mean straight leg raise in the participants without LBP was significantly greater than the mean straight leg raise in the LBP group (*p* < 0.05)
**Ringheim et al., 2015 **[62] **Norway**	To investigate muscle activation level and variability, and postural control during 15 min of prolonged standing and the effect of prolonged standing on neuromuscular control, postural sway and strength between participants with and without chronic LBP	LBP *n* = 17, without LBP *n* = 21 Age LBP: 39.0 ± 5.4 years, without LBP: 40.2 ± 5.4 years Sex: LBP 10 female, 7 male, without LBP 13 female, 8 male Pain: 5.0 ± 1.7, measured by the Numerical pain rating scale (0–10) Disability: 21.1% ± 7.8%, measured by the Oswestry disability index (0–100%)	EMG signals detected bilaterally from the erector spinae, gluteus medius, rectus abdominus and external oblique muscles Ground reaction forces recorded for each foot separately using two force plates	The relative muscle activation level at the start and during the prolonged standing was higher in the patients with chronic LBP for all muscles, except for the gluteus medius (*p* = 0.19) Patients with LBP made significantly more body weight shifts (median 47, interquartile range 8 to 89) compared to participants without LBP (median 3, interquartile range 0 to 21, *p* = 0.03) and had increased postural sway values (center of pressure speed (mm/s) in medio–lateral direction: patients with LBP, median 20.5, interquartile range 14.3 to 27.4, participants without LBP, median 12, interquartile range 10.1 to 21.6, *p* = 0.03; center of pressure speed (mm/s) in anterior–posterior direction: patients with LBP, median 31.1, interquartile range 15 to 40.7, participants without LBP, median 13.5, interquartile range 11.1 to 29.5, *p* = 0.01). The perceived exertion after standing from pre to post standing was significantly greater in the patients with LBP (median 13.5, interquartile range 11.5 to 15) compared to participants without LBP (median 7, interquartile range 7 to 9, *p* < 0.01)
**Shojaei et al., 2017 **[63] **United States**	To investigate differences in the low back mechanical environment, using measures of trunk kinematics, between females with and without acute LBP	LBP *n* = 19, without LBP *n* = 19 Age LBP: 58.0 ± 9.0 years, without LBP: 56.0 ± 9.0 years Sex: 100% female	Kinematics tracked using wireless Inertial Measurement Units (pelvic and thoracic ranges of rotation, lumbar range of flexion)	Pelvic range of rotation was larger in patients with LBP (61.6° ± 12°) than asymptomatic participants (43.4° ± 14.5°, *p* < 0.05) and was larger in tasks with fast pace (56.7 ± 15.2°) than self-selected pace (48.3 ± 16°, *p* < 0.05) Peak angular velocity of lumbar flexion was higher in asymptomatic participants (94.7 ± 26 ºS^−1^) than in patients with LBP (65.5 ± 31ºS^−1^, *p* = 0.006) and was higher during the forward bending (84.7 ± 33ºS^−1^) than the backward return (78 ± 28 ºS^−1^, *p* = 0.004) phase of the motion
**Adhia et al., 2016 **[68] **New Zealand**	To determine if the sacroiliac joint positive individuals demonstrate significantly different innominate kinematic measures (movement pattern, ROM and trends of rotation) in the hip abductor external rotation test when compared with sacroiliac joint negative individuals	Total *n* = 122 Age LBP: sacroiliac joint negative individuals 30.2 ± 9.8 years, right sacroiliac joint positive individuals 35.1 ± 10.6 years, left sacroiliac joint positive individuals 35.7 ± 7.2 years, bilateral sacroiliac joint positive individuals 28.6 ± 10.3 years Pain: sacroiliac joint negative individuals 7.6 ± 11.4, right sacroiliac joint positive individuals 7.1 ± 6.6, left sacroiliac joint positive individuals 5.9 ± 6.1, bilateral sacroiliac joint positive individuals 5.4 ± 5.0, measured by the Visual analog scale (0–10) Disability: sacroiliac joint negative individuals 12.0% ± 8.8, right sacroiliac joint positive individuals 12.1% ± 8.6, left sacroiliac joint positive individuals 11.1% ± 10.7, bilateral sacroiliac joint positive individuals 14.1 ± 5.1, measured by the Oswestry disability index (0–100%)	Angular displacement of the innominate angle between the neutral hip test position and maximum prone hip abduction and external rotation test assessed using the Polhemus™ electromagnetic palpation digitization technique	A significant positive association was demonstrated between patients with LBP and sacroiliac pain and the unilateral innominate movement pattern (right sacroiliac joint positive versus sacroiliac joint negative individuals *p* < 0.001, left sacroiliac joint positive versus sacroiliac joint negative individuals *p* = 0.003, bilateral sacroiliac joint positive versus sacroiliac joint negative individuals *p* = 0.001) The sacroiliac joint positive individuals exhibited significantly different innominate trends of rotation when compared with the sacroiliac joint negative individuals (right prone hip abduction and external rotation test, right innominate trend, left sacroiliac joint positive individuals have a significant difference compared to sacroiliac joint negative individuals, *p* < 0.017, right prone hip abduction and external rotation test, left innominate trend, left sacroiliac joint positive individuals, bilateral sacroiliac joint positive individuals and right sacroiliac joint positive individuals have a significant difference compared to sacroiliac joint negative individuals, *p* < 0.017, left prone hip abduction and external rotation test, left innominate trend, left sacroiliac joint positive individuals and bilateral sacroiliac joint positive individuals have a significant difference compared to sacroiliac joint negative individuals, *p* < 0.017, left prone hip abduction and external rotation test, right innominate trend, right sacroiliac joint positive individuals have a significant difference compared to sacroiliac joint negative individuals, *p* < 0.017, left prone hip abduction and external rotation test, sagittal innominate trend, right sacroiliac joint positive individuals, left sacroiliac joint positive individuals and bilateral sacroiliac joint positive individuals have a significant difference compared to sacroiliac joint negative individuals, *p* < 0.017)
**Choi et al., 2022 **[66] **South Korea**	To compare the length of the hip flexor and the muscle activity of the gluteus maximus during hip extension in people with or without non specific chronic LBP	LBP *n* = 16, without LBP *n* = 17 Age LBP: 28.5 ± 3.8 years, without LBP 28.6 ± 5.4 years Sex: LBP 6 female, 10 male, without LBP 6 female, 11 male Pain: 5.3 measured by the numeric pain rating scale (0–10) Disability: 14.6 measured by the Korean Oswestry disability index (0–45)	Hip flexor length ROM was recorded using a digital inclinometer during modified Thomas test. Muscle activity of the gluteus maximus was measured using a Delsys-Trigno wireless EMG system (Delsys Inc., Boston, MA, USA)	Hip flexor length: Dominant LBP 18.2 ± 6º, without LBP 6.1 ± 8.3º, *p* < 0.05; Non-dominant LBP 18.6 ± 5.5º, without LBP 7.1 ± 7.3º, which was significantly higher in the LBP group than in the non-LBP group (*p* < 0.05). Muscle activity: gluteus maximus (%MVIC) LBP 14 ± 5.8, without LBP 22.2 ± 6.6, which was significantly lower in the LBP group than in the non-LBP group *p* <; 0.05
**Hamill et al., 2009 **[59] **United States**	To examine the joint stiffness from the moment-joint angle relationship of the hip in patients with LBP and resolved LBP and to compare with participants without LBP	LBP *n* = 11, resolved LBP *n* = 11, without LBP n:11 Age LBP: 35.7 ± 11.0 years, resolved LBP: 32.6 ± 9.4 years, without LBP: 29.9 ± 8.5 years Pain: 8.3 ± 1.4, measured by the Visual analog scale (1–10) Disability: 7.9 ± 6.2, measured by the Modified Oswestry disability index (0–100%)	Cameras and force transducers of the treadmill interfaced with the same microcomputer 3D motion analysis software used to filter kinematic and stiffness of the hip joints	There was no significant difference between groups for hip stiffness, peak extensor moment and hip ROM
**Park et al., 2012 **[69] **South Korea**	To compare different ROM on kinematic changes of the pelvic joints during axial trunk rotation activities in standing	LBP *n* = 19, without LBP *n* = 19 Age: 68. 9 ± 5.5 years Sex: LBP 15 female, 4 male, without LBP 11 female, 8 male	Kinematic data of joint angles recorded and processed using six digital cameras capturing 3D full body kinematic motion sampling at 120 Hz Shoulder and pelvic ROM assessed using reflective markers during axial trunk rotation test	There was a significant decrease in the pelvic ROM in patients with LBP (82.1º ± 23.2º) compared with asymptomatic participants (100.8º ± 26.5º, *p* = 0.02)
**Vatandoost et al., 2022 **[67] **Iran**	To compare the strength (gluteal and hamstring) and flexibility (hip flexor and hamstring) of key muscle groups between an active extension pattern subgroup and healthy controls	LBP *n* = 16, without LBP *n* = 16 Age LBP: 23.3 ± 2.7 years, without LBP 22.9 ± 1.9 years Sex: 100% female Pain: 0.9 ± 0.9 measured by the Visual analog scale (0–10)	Gluteus maximus, gluteus medius and hamstring strength was measured by dynamometer Hip flexor and hamstring flexibility was assessed using a universal two-arm goniometer Hip flexor ROM was measured using the Thomas test	The LBP group displayed significantly lower strength of all muscles analyzed (*p* < 0.007, large effect size), with the exception of gluteus medius (Gluteus maximus strength: control group 0.5kgf/kg ± 0.08, LBP group 0.4 kgf/kg ± 0.1, *p* = 0.002; Hamstring strength control group 0.5 kgf/kg ± 0.1, LBP group 0.4 kgf/kg ± 0.1, *p* = 0.001). There was no ROM difference between LBP and without LBP group
**Vogt et al., 2003** [65] **Germany**	To examine changes in the hip extensor activation patterns in patients with chronic LBP during walking	LBP *n* = 17, without LBP *n* = 16 Age LBP: 36.3 ± 2.1 years, without LBP: 33.7 ± 3.1 years Sex: 100% male Pain: 3.0 ± 5.3, measured by the Visual analog scale (0–10) Disability: 26.3%, range 24–48, measured by the Oswestry disability index (0–100%)	Hip joint ROM assessed using an electronic goniometer in the sagittal plane and EMG activity of the gluteus maximus and biceps femoris muscles recorded unilaterally during treadmill walking at 1.25 m/s	Significant differences for the hip joint ROM (38.3 ± 9.11º vs 25.2 ± 7.91º, *p* < 0.01) and stride time (1.1 ± 0.1 s vs 1 ± 0.1 s, *p* < 0.01) were found between asymptomatic participants and patients with LBP Significant EMG onset differences were found in the assessed biceps femoris and gluteus maximus muscles, the LBP group started late compared to the asymptomatic participants (*p* < 0.01) Additionally, the analysis revealed a significantly prolonged EMG activity of the gluteus maximus muscle in the LBP group (*p* < 0.01) Phase shifts identified by the cross-correlation calculations confirmed the pre-matured EMG activity of the biceps femoris (*p* < 0.01) and gluteus maximus muscles (*p* < 0.01) in the patients with LBP
**Busssey et al., 2016 **[16] **New Zealand**	To examine the effect of prolonged standing on gluteus medius muscle coactivation and to observe whether the gluteus medius muscle coactivation over time was related to the development of LBP in elite female field hockey players with and without LBP	LBP *n* = 14, without LBP *n* = 25 Age LBP: 19.3 ± 1.4 years, without LBP: 20.0 ± 1.7 years Sex: 100% female Pain: 4.9 ± 6.5, measured by the Visual analog scale (0–10) Disability: 5.9 ± 4.8%, measured by the Oswestry disability index (0–100%)	Gluteus medius muscle strength measured using a force transducer during a restricted hip abduction test Active hip abduction ROM measured using a Polhemus Fastrak system operating at 30 Hz Gluteus medius muscle surface EMG data recorded continuously using two electrodes	There were statistically significant between-group differences in the hip abduction ROM (LBP group left side 66.2 ± 5º, right side 66.7 ± 5.1º, without LBP group left side 70 ± 6.6º, right side 69.7 ± 6.3, *p* = 0.020) ANOVA showed a significant time effect on both gluteus medius muscle coactivation (*p* = 0.003). Significant differences were found between the groups at 10 (*p* = 0.027), 20 (*p* = 0.008), 50 (*p* = 0.013), 60 (*p* = 0.009), and 70 (*p* = 0.012) minutes There was a significant stand time effect, as well as a significant group x time interaction (*p* < 0.001). The LBP group had a higher pain score on commencement (first 10 min mark) of the prolonged stand and steadily increased over the entirety of the stand, while the group without LBP displayed a relatively stable pain score (*p* < 0.001)
**Crosbie et al., 2013 **[56] **Australia**	To compare temporal and spatial coordination of rotations in the transverse and frontal planes of the low thoracic and lumbar spinal regions during walking at preferred and fast speeds in participants with and without a history of recurrent LBP	LBP *n* = 19, without LBP *n* = 19 Age LBP: 34.0 ± 13.3 years, without LBP: 28.6 ± 5.4 years Sex: LBP 12 female, 7 male, without LBP 13 female, 6 male Pain: 2.8 ± 2.2, measured by the Visual analog scale (0–10) Disability: 4.2 (0–19 [range]), measured by the Roland Morris disability questionnaire (0–24)	Trunk segmental movements recorded using a multisensor, 6-df electromagnetic tracking device ROM of trunk, pelvis, and hip joints assessed using a digital anatomical landmark	For ROM, the only between-group difference was a smaller pelvic side flexion at preferred speed in patients with LBP (without LBP 8.9 ± 2.9º, LBP 7.3 ± 2º, *p* < 0.05) Walking speed in patients with LBP was smaller than in participants without LBP (*p* = 0.02) Considering the movement coordination between segments, the phase lag at intersegmental lower thoracic and lumbar for axial rotation was significantly less in patients with LBP (21% ± 14.7) than in participants without LBP (2.8% ± 9.8) at a preferred speed (*p* < 0.05). Phase lag at intersegmental side flexion and axial rotation was significantly less in patients with LBP (- 9% ± 9.3) than in participants without LBP (0% ± 11.2) at a preferred speed (*p* < 0.05) The movements of the pelvis during axial rotation were significantly greater in patients with LBP (preferred speed 0.99 ± 0.01 m/s, fast speed 0.98 ± 0.01 m/s) than in participants without LBP (preferred speed 0.98 ± 0.05 m/s, fast speed 0.96 ± 0.05 m/s, *p* < 0.05)
**Haddas et al., 2019 **[57] **United States**	To determine the effects of volitional preemptive abdominal contraction and recurrent LBP on trunk mechanics and neuromuscular control during an asymmetrical 1-m box-lift task	LBP *n* = 32, without LBP *n* = 37 Age LBP: 21.6 ± 2.2 years, without LBP: 20.4 ± 4.2 years Pain: in the same day 2.0 ± 0.9, in the last week 3.9 ± 1.5, worst pain in the last week 4.8 ± 1.9, measured by the Visual analog scale (1–10)	EMG of external oblique and gluteus maximus muscle, 3D spine and pelvic joint kinematics using a Vicon motion during asymmetric lifting	The LBP group demonstrated reduced EMG activity for the external oblique muscle (80.6 ± 5.5% maximum voluntary contraction) at the initial position of the lifting compared to participants without LBP (92.9 ± 5.6% maximum voluntary contraction, *p* = 0.01). Furthermore, the LBP group performed the lift with smaller gluteus maximus muscle activity at the initial position of lifting (*p* = 0.047) The LBP group exhibited greater hip flexion angle (LBP group 23.1º ± 8.4º, without LBP group 18.4 ± 7.5º, *p* = 0.008) and smaller hip abduction angle at the final position of the lifting (LBP group -2.9 ± 6.9º, without LBP group 2.3 ± 6.1º, *p* = 0.001)
**Hicks et al., 2021 **[71] **Unites States**	To investigate whether clinically relevant subgroups of older adults with chronic LBP could be identified on the basis of the presence of potentially modifiable hip impairments using latent variable mixture modeling and to examine the relationship between these subgroups and key outcomes, including performance-based mobility function, over a period of 12 months	Total *n* = 250 Age: 69.7 ± 6.8 years Sex: 128 female, 122 male Disability: Subgroup 1 (strong and nonsymptomatic 21.6 (12.6), Subgroup (weak and nonsymptomatic) 26.0 (16.4), Subgroup 3 (weak and symptomatic) 38.8 (15.8), measured by the Quebec Disability Questionnaire (0–100)	The muscle strength of hip flexion, abduction, adduction, internal rotation, external rotation, and extension was assessed using a handheld dynamometer A goniometer was used to measure hip flexion, abduction, adduction, and extension ROM, and an inclinometer was used to measure hip internal and external rotation The Modified Thomas Test, straight leg raise, and Ober Test were used to quantify hip flexor, hip extensor, and iliotibial band flexibility, respectively	Subgroup 1 was stronger across all hip strength measurements than subgroups 2 and 3 (*p* <; 0.001); although subgroup 2 was stronger than subgroup 3 on all strength measurements, except for hip flexion (*p* <; 0.01), the differences were less pronounced (Flexion Subgroup 1 0.65 kg/BMI (0.23), Subgroup 2 0.38 kg/BMI (0.15), Subgroup 3 0.33 kg/BMI (0.12); Abduction Subgroup 1 0.37 kg/BMI (0.08), Subgroup 2 0.21 kg/BMI (0.06), Subgroup 3 0.18 kg/BMI (0.06); Adduction Subgroup 1 0.41 kg/BMI (0.09), Subgroup 2 0.22 kg/BMI (0.06), Subgroup 3 0.18 kg/BMI (0.07); Internal rotation Subgroup 1 0.34 kg/BMI (0.08), Subgroup 2 0.17 kg/BMI (0.05), Subgroup 3 0.14 kg/BMI (0.05); External rotation Subgroup 1 0.42 kg/BMI (0.11), Subgroup 2 0.22 kg/BMI (0.07), Subgroup 3 0.18 kg/BMI (0.07); Extension Subgroup 1 0.35 kg/BMI (0.13), Subgroup 2 0.18 kg/BMI (0.08), Subgroup 3 0.14 kg/BMI (0.07) Regarding ROM, subgroups 1 and 2 had higher hip flexion than subgroup 3 (Subgroup 1 93.4º (7.3), Subgroup 2 93.2º (2.9), Subgroup 3 90.2º (8.2), *p* <; 0.05) and subgroup 1 showed lower internal rotation than subgroup 2 (Subgroup 1 21.0º (9.8), Subgroup 2 24.8º (10.8), *p* = 0.02) and higher external rotation than subgroup 3 (Subgroup 1 31.8º (9.9), Subgroup 3 27.7º (9.6), *p* = 0.02)
**Van Wingerden et al., 2008 **[64] **Netherlands**	To determine whether subcategories of patients with LBP could be distinguished by motion characteristics of the pelvis and lumbar spine	LBP *n* = 22, pelvic girdle pain *n* = 29, without LBP *n* = 53 Age LBP: 36.0 ± 9.0 years, pelvic girdle pain: 33.0 ± 5.0 years, without LBP: 25.0 ± 9.0 years Sex:100% female Pain: LBP 55.0 ± 25.0 mm, pelvic girdle pain 54.0 ± 24.0 mm for actual pain, LBP 32.0 ± 20.0 mm, pelvic girdle pain 28.0 ± 16.0 mm for minimal pain, LBP 86.0 ± 15.0 mm, pelvic girdle pain 89.0 ± 11.0 mm for maximal pain, measured by the Visual analog scale (0-100 mm) Disability: LBP 45.0 ± 15.0, pelvic girdle pain 61.0 ± 10.0, measured by the Quebec Disability Scale (0–100)	Motion recorded in the sagittal plane using a video-camera Abduction and adduction strength of the hips measured using a handheld dynamometer	Patients with pelvic girdle pain showed a significant backward tilt of the pelvis (7 ± 4°, *p* < 0.001) compared with the participants without LBP ROM to flexion from the upright position of trunk was significantly decreased in patients with LBP (81 ± 23º) and in the pelvic girdle pain group (83 ± 28º) compared to the participants without LBP (116 ± 14º, *p* < 0.001) ROM to flexion from the upright position of pelvis was significantly decreased in the pelvic girdle pain group (37 ± 19º) compared to the participants without LBP (56 ± 13º, *p* < 0.001) ROM to flexion from the upright position of lumbar spine was significantly decreased in patients with LBP (30 ± 16º) and in the pelvic girdle pain group (47 ± 14º) compared to the participants without LBP (60 ± 9º, *p* < 0.001) Abduction strength was significantly different between the patients with LBP (245 ± 83N) and the pelvic girdle pain group (146 ± 74N, *p* < 0.001) Adduction strength was significantly different between the patients with LBP (176 ± 55N) and the pelvic girdle pain group (83 ± 51N, *p* < 0.001)
**Ciesielska et al., 2015 **[55] **Poland**	To detect alterations in the hip strategy manifested by differences in balance parameters and rectus femoris and gluteus maximus muscular activity in people with a previous episode of LBP	LBP *n* = 9, without LBP *n* = 10 Age LBP: 48.0 ± 9.0 years, without LBP: 30.0 ± 10.7 years Sex: LBP 6 female, 3 male, without LBP 8 female, 2 male	Stability of the upright posture and changes of the gluteus maximus and rectus femoris muscular activity in the lower extremities obtained from a surface EMG and a balance platform	Patients with LBP showed differences in the number of fluctuations for the gluteus maximus muscle in the position of eyes opened and eyes closed (number of fluctuations, gluteus maximus without LBP eyes opened 11.9 ± 6.3, LBP group affected limb 6.2 ± 6.9, unaffected limb 7.4 ± 8.2, without LBP eyes closed 10.1 ± 6, LBP group affected limb 5.2 ± 6.8, unaffected limb 7.3 ± 7.2) In the eyes opened and eyes closed positions, a significantly higher average amplitude was recorded from the activity of the rectus femoris muscle on the unaffected side (number of fluctuations, rectus femoris without LBP eyes opened 11.6 ± 7.4, LBP group unaffected limb 5.2 ± 7.1, number of fluctuations, rectus femoris without LBP eyes closed 9.5 ± 6.4, LBP group unaffected limb 4.8 ± 5.6, *p* < 0.05)
**Jones et al., 2012 **[61] **United States**	To characterize the movement patterns of participants with and without LBP, by comparing their automatic postural responses elicited by multi-directional support surface translations	LBP *n* = 16, without LBP *n* = 16 Age LBP: 33.9 ± 6.2 years, without LBP: 33.5 ± 9.0 years Sex: 50% female Pain: 3 (0–7), measured by the Numerical pain rating scale (0–10) (values presented as median [range]) Disability: 19.8 ± 8.8, measured by the Oswestry disability index (0–100)	Two force plates mounted within the moveable platform driven by electromechanical motors A 3-camera passive marker system was used to collect 3-dimensional body kinematic data Surface EMG of the trunk and lower limb muscles	For the peak torque latencies, temporal differences were significant between the LBP and without LBP groups. The LBP group demonstrated earlier sagittal peak joint torques at the hips compared to the group without LBP (LBP group: left, *p* = 0.03; right, *p* = 0.04)

*ROM* Range of motion, *LBP* low back pain, *EMG* electromyography, *N* newtons, *CI* confidence interval, *Kpa* kilopascal, *s* seconds, *Nm/kg* newton meter per kilogram, º*S*^*−1*^ degree per second, *cm* centimeter, *ms* millisecond, *mm/s* millimeters per second, *m/s* meters per second, *3D* three dimensions, *s* seconds, º degree, *Hz* hertz, *kgf/kg* kilograms force per kilogram mass, *MVIC* maximum voluntary isometric contraction, *Kg/BMI* kilogram normalized to body mass index, *μV* microvolt

^a^All values are presented as mean ± standard deviation, unless stated

#### Range of motion

Biomechanical assessments of range of motion were performed using goniometer [26, 58, 60, 65, 67, 71], inclinometer [22, 24, 25, 28–30, 66], and Thomas [23, 29, 66, 67, 71], Ober [71] and Straight leg raise test [58, 71]. The most investigated movement was total hip rotation[22, 25, 28, 30], followed by internal rotation [24, 26, 30, 71] and external hip rotation [22, 26, 71]. The results suggested a reduction in the total hip range of motion in patients with acute and chronic LBP [22, 25, 28, 30] and a reduction in internal rotation [24, 26, 30] and external hip rotation in patients with subacute and chronic LBP [22, 26]. Two studies identified a reduced range of motion between the dominant and non-dominant lower limbs [30] and between men and women with subacute and chronic LBP [26]. Only one study [63] showed that patients with acute LBP had a higher range of hip rotation compared to healthy individuals.

#### Strength

Biomechanical strength assessments were performed using the manual muscle test [32, 54], pressure meter [31, 33], force transducer [16] and hand-held dynamometer [34, 64, 67, 70, 71]. The main movement tested was hip abduction [16, 32–34, 64, 67, 70, 71]. The results showed that the hip abductor and extensor muscles were weaker in patients with acute, subacute, and chronic LBP compared to healthy individuals [31–34].

#### Kinematics

Biomechanical kinematic assessments were performed using 3-dimensional systems [35, 37, 39, 41, 42, 45, 46, 57, 59, 69], force platform [36, 37, 44, 55, 62, 70], cameras [38, 40, 43, 44, 64] and sensors [49, 50, 56, 60, 63, 68]. Hip movements were assessed during functional activities such as sit-to-stance-to-sit [36], sit-to-stand [37, 49], walking [46, 56, 59, 65], among others [35, 40, 44, 49, 57, 60, 64, 68, 69]. The results showed that during sit-to-stand, patients with chronic LBP had limited trunk flexion [37] and patients with subacute LBP had reduced task execution time [36, 49]. During walking, patients with chronic LBP showed a reduction in gait speed [46, 56] and step distance [65] compared to healthy individuals.

#### Electromyography

Electromyography was used to assess the gluteus maximus [17, 18, 27, 51, 53–55, 57, 65, 66], hamstrings [17, 18, 52, 53, 58, 65] and gluteus medius [16, 27, 54, 62] muscles. Muscle activation was performed in various activities, from exercise [27, 51] to functional activities such as sit-to-stand and stand-to-sit [52, 53] and standing [16, 55, 62]. The results were very different between the studies as the muscles were tested in different ways. Two studies tested the gluteus medius and maximus muscles during exercise [27, 51] and found no differences between patients with chronic LBP and healthy individuals. Two other studies [52, 53] tested the hamstring muscles in similar positions (semi-sitting position [53] and sit-to-stand and stand-to-sit [52]) and obtained different results. One study [53] evaluated the semi-sitting position during an isometric contraction and found no difference between patients with chronic LBP and healthy individuals, while other study [52] evaluated the sit-to-stand and stand-to-sit dynamically and observed decreased activation of the time to peak in the hamstring muscles of patients with chronic LBP compared to healthy individuals.

## Discussion

The objective of this systematic review was to summarize the results of observational studies that performed biomechanical assessments in patients with non-specific LBP. The 54 studies included in the review used the outcomes range of motion, kinematics, strength, and electromyography for biomechanical assessment. The most common assessments were range of motion and kinematics. Patients with LBP, regardless of the duration of symptoms, showed a significant reduction in hip range of motion, especially total hip rotation [22, 25, 28, 30] and internal hip rotation [19, 24, 26, 30], even with the use of different assessment tools [19, 24–26, 28–30]. Range of motion can be assessed in different ways. The goniometer [26, 58, 60, 65, 67, 71] and inclinometer [22, 24, 25, 28–30, 66] are the most routine, despite having a measurement error between 3 and 5 degrees [73, 74]. Thus, there may be differences in range of motion, such as between dominant and non-dominant lower limbs [30] and between men and women with subacute and chronic LBP [26], that are not greater than the measurement error in studies assessing this outcome. However, the studies included in this systematic review found a significant reduction in total hip rotation in a variety of patients with LBP from participants of various sports [28, 30] to helicopter pilots[25] compared to healthy individuals. Patients with subacute and chronic LBP showed a significant reduction in internal hip rotation compared to healthy individuals [24, 26, 30], and one study showed an association between LBP and reduced internal hip rotation [19].

Kinematics has been extensively studied in patients with LBP, especially during functional activities such as sit-to-stand [36, 37, 49], walking [38, 56, 65] and lifting [35, 40]. Although the current kinematic assessment aims to assess common day-to-day movements [75], no studies have been found that performed kinematic assessment during everyday functional activities that generate pain in patients with LBP, such as putting on shoes or pants [76]. In general, patients with chronic LBP showed a reduction in the execution time of functional activities and range of motion, which indicates that they use different strategies than healthy individuals to perform the same functional activities [77]. The human movement system has the ability to adapt and use new strategies in the short and long term [75]. These strategies adopted by patients with LBP may be the result of motor adaptations to avoid painful movements during the execution of tasks [78, 79]. This was observed in a previous systematic review [80], that showed “moderate” strength of evidence for reduction of gait preference velocity and “high” strength of evidence for decrease in stride distance in patients with LBP compared to healthy individuals.

The gluteus maximus [17, 18, 27, 51, 53, 54] and hamstring muscles [17, 18, 52, 53] were the most evaluated in studies that used electromyography as a form of assessment. In addition to being the main extensor muscles of the hip [81], they are superficial, which facilitates electromyographic assessment [51]. During the electromyographic assessment, standardization is recommended such as using more than one channel per muscle group and normalizing the value obtained by the maximum voluntary contraction [82, 83]. However, in patients with LBP, the recommendation is to perform normalization by submaximal voluntary contraction, reducing the chance of interference from the pain intensity [17, 52]. The studies included in this systematic review [27, 51] did not report the patients' pain intensity, therefore it is not possible to know if there was interference during normalization. However, the gluteus maximus and hamstring muscles had greater electromyographic activation in patients with chronic LBP compared to healthy individuals during the prone hip extension task [17], which may indicate that patients with chronic LBP have difficulty maintaining a stable pelvic lumbar region [17, 84].

In this systematic review, 9 studies [16, 31–34, 64, 67, 70, 71] evaluated the strength of hip muscles in patients with LBP. Despite the good reliability between the hand-held dynamometer and the isokinetic dynamometer [85] and the difference in cost between the devices, the hand-held dynamometer is still inaccessible to many health professionals [86–88]. The cost of the manual dynamometer may be one of the reasons why the included studies assessed strength using a manual muscle test [32] and pressure meter [31, 33]. The results of this assessment corroborate a recent systematic review [15], in which patients with acute, subacute, and chronic LBP presented weakness of the abductor [32–34, 71] and extensor muscles [31, 34, 67, 71] of the hip compared to healthy individuals.

Although LBP affects millions of people worldwide [5, 6] and some studies seek to understand how the hip behaves in the presence of LBP [14–19], this is the first systematic review that summarizes the main findings of biomechanical hip assessments in patients with LBP, considering the type of assessment, the objective of each study, and its result. The results of this systematic review allow an overview of what is expected in the hip assessment of patients with LBP, directing clinicians to more accurate assessments and researchers to new studies that investigate the causes of LBP in a specific population or risk factors in an asymptomatic population. Future research may determine how much the biomechanical outcomes of the hip can be modified during the treatment of patients with LBP, as this question remains unanswered [89]. On the other hand, the heterogeneity of the biomechanical assessments and styles of reporting presented a challenge in this systematic review. Although the methodological quality of the included studies was moderate or high, the results were not always presented clearly.

## Conclusion

The studies that evaluated the hip biomechanics of patients with LBP are of moderate and high methodological quality. Range of motion is lower in the total, internal, and external hip rotation movements of patients with LBP compared to healthy individuals. The strength of the hip abductor and extensor muscles is lower in patients with LBP compared to asymptomatic individuals. In the kinematic assessment, patients with LBP adopt strategies to reduce speed and change hip flexion movements compared to asymptomatic individuals during functional activities. Patients with LBP submitted to electromyographic assessment presented shorter activation time of the hip muscles and greater amplitude of electromyographic activity compared to healthy individuals. Therefore, greater attention should be given to hip assessment and management during the treatment of these patients.

### Supplementary Information

**Supplementary Material 1.**

## Data Availability

All datasets used during the current study are available from the corresponding author on reasonable request.

## Abbreviations

ROM: Range of motion
LBP: Low back pain
EMG: Electromyography
PRISMA: Preferred Report Items for Systematic Review and Meta-Analyses
PROSPERO: International Prospective Register of Systematic Reviews
NR: Not reported
N: Newtons
CI: Confidence interval
KPa: Kilopascal
s: Seconds
Nm/kg: Newton meter per kilogram
ºS^−^^1^: Degree per second
ms: Millisecond
mm/s: Millimeters per second
m/s: Meters per second
3D: Three dimensional
