# Supplementary material for: Hip biomechanics in patients with low back pain, what do we know? A systematic review
Source: BMC Musculoskelet Disord. 2024 May 28;25:415. doi: 10.1186/s12891-024-07463-5 (PMC11131240; doi:10.1186/s12891-024-07463-5)
Supplement: Supplementary file 1 — Supplementary Material 1. [file 12891_2024_7463_MOESM1_ESM.docx]

**SEARCH STRATEGY**

**Study:** Biomechanical evaluation of the hip in patients with chronic low back pain - Systematic review of observational studies.

**Authors:** Gustavo Zanotti Pizol, Gisela Cristiane Miyamoto e Cristina Maria Nunes Cabral.

**Objective of the review:** Systematically review observational studies that used biomechanical analysis of the hip in patients with low back pain and verify the methodological quality of the included studies.

| **Databases** | **Name** | **Date** |
| --- | --- | --- |
| PUBMED | Gustavo | 02/22/2024 |
| EMBASE | Gustavo | 02/22/2024 |
| CINAHL | Gustavo | 02/22/2024 |
| SPORTDiscus | Gustavo | 02/22/2024 |

PUBMED

| **Search performed** |  |
| --- | --- |
| **Database** | PUBMED |
| **Account** |  |
| **Limits** |  |
| **Hits** |  |
| **Search** | ***Study design***  *Observational Study*  #1 ((((((((((((((((((((((((((((((((((((((((((((((((((("Cohort Study") OR ("Studies, Cohort")) OR ("Study, Cohort")) OR ("Concurrent Studies")) OR ("Studies, Concurrent")) OR ("Concurrent Study")) OR ("Study, Concurrent")) OR ("Closed Cohort Studies")) OR ("Cohort Studies, Closed")) OR ("Closed Cohort Study")) OR ("Cohort Study, Closed")) OR ("Study, Closed Cohort")) OR ("Studies, Closed Cohort")) OR ("Analysis, Cohort")) OR ("Cohort Analysis")) OR ("Analyses, Cohort")) OR ("Cohort Analyses")) OR ("Historical Cohort Studies")) OR ("Cohort Study, Historical")) OR ("Historical Cohort Study")) OR ("Study, Historical Cohort")) OR ("Cohort Studies, Historical")) OR ("Studies, Historical Cohort")) OR ("Incidence Studies")) OR ("Incidence Study")) OR ("Studies, Incidence")) OR ("Study, Incidence")) OR ("Case-Control Study")) OR ("Studies, Case-Control")) OR ("Study, Case-Control")) OR ("Case-Comparison Studies")) OR ("Case Comparison Studies")) OR ("Case-Comparison Study")) OR ("Studies, Case-Comparison")) OR ("Study, Case-Comparison")) OR ("Case-Compeer Studies")) OR ("Studies, Case-Compeer")) OR ("Case-Referrent Studies")) OR ("Case Referrent Studies")) OR ("Case-Referrent Study")) OR ("Studies, Case-Referrent")) OR ("Study, Case-Referrent")) OR ("Case-Referent Studies")) OR ("Case Referent Studies")) OR ("Case-Referent Study")) OR ("Studies, Case-Referent")) OR ("Study, Case-Referent")) OR ("Case-Base Studies")) OR ("Case Base Studies")) OR ("Studies, Case-Base")) OR ("Case Control Studies")) OR ("Case Control Study") Hits: 3.068.049   *Low back pain*  #2 ((((((((((((((((((((((((((((("Low back pain") OR ("Back Pain, Low")) OR ("Back Pains, Low")) OR ("Low Back Pains")) OR ("Pain, Low Back")) OR ("Pains, Low Back")) OR (Lumbago)) OR ("Lower Back Pain")) OR ("Back Pain, Lower")) OR ("Back Pains, Lower")) OR ("Lower Back Pains")) OR ("Pain, Lower Back")) OR ("Pains, Lower Back")) OR ("Low Back Ache")) OR ("Ache, Low Back")) OR ("Aches, Low Back")) OR ("Back Ache, Low")) OR ("Back Aches, Low")) OR ("Low Back Aches")) OR ("Low Backache")) OR ("Backache, Low")) OR ("Backaches, Low")) OR ("Low Backaches")) OR ("Low Back Pain, Postural")) OR ("Postural Low Back Pain")) OR ("Low Back Pain, Posterior Compartment")) OR ("Low Back Pain, Recurrent")) OR ("Recurrent Low Back Pain")) OR ("Low Back Pain, Mechanical")) OR ("Mechanical Low Back Pain")  **Hits: 53.058**    *Hip*  #3 ((((((((Hip) OR (Hips)) OR ("Hip Joints")) OR ("Joint, Hip")) OR ("Joints, Hip")) OR ("Acetabulofemoral Joint")) OR ("Acetabulofemoral Joints")) OR ("Joint, Acetabulofemoral")) OR ("Joints, Acetabulofemoral")  **Hits: 205.188**    ***Avaliação biomecânica***  #4 (((((((((((((((((((((((((((((((((((((((((((((((((((((((((Electromyographies) OR (Electromyography)) OR ("Surface Electromyography")) OR ("Electromyographies, Surface")) OR ("Electromyography, Surface")) OR ("Surface Electromyographies")) OR (Electromyogram)) OR (Electromyograms)) OR ("Dynamometer, Muscle Strength")) OR ("Dynamometers, Muscle Strength")) OR ("Muscle Strength Dynamometers")) OR ("Strength, Muscle")) OR ("Arthrogenic Muscle Inhibition")) OR ("Arthrogenic Muscle Inhibitions")) OR ("Inhibition, Arthrogenic Muscle")) OR (Biomechanics)) OR ("Muscle Inhibition, Arthrogenic")) OR ("Phenomena, Biomechanical")) OR ("Biomechanic Phenomena")) OR ("Biomechanic Phenomenas")) OR ("Phenomena, Biomechanic")) OR ("Phenomenas, Biomechanic")) OR ("Mechanobiological Phenomena")) OR ("Phenomena, Mechanobiological")) OR (Kinematics)) OR ("Performance Test")) OR ("Endurance Test")) OR ("Functional Performance, Physical")) OR ("Functional Performances, Physical")) OR ("Performance, Physical Functional")) OR ("Performances, Physical Functional")) OR ("Physical Functional Performances")) OR ("Functional Performance")) OR ("Functional Performances")) OR ("Performance, Functional")) OR ("Performances, Functional")) OR ("Physical Performance")) OR ("Performance, Physical")) OR ("Performances, Physical")) OR ("Physical Performances")) OR ("muscle activation")) OR ("Development, Muscle")) OR ("Muscular Development")) OR ("Development, Muscular")) OR (Myogenesis)) OR (Myofibrillogenesis)) OR ("Range of Motion")) OR ("Joint Range of Motion")) OR ("Joint Flexibility")) OR ("Flexibility, Joint")) OR ("Range of Motion")) OR ("Passive Range of Motion")) OR (“Joint motion”)) OR ("Joint Range of Motion")) OR ("Joint Flexibility")) OR ("Flexibility, Joint")) OR ("Range of Motion")) OR ("Passive Range of Motion")  **Hits: 604.201**  ***Final connections***  #5 (((#1) AND (#2)) AND (#3)) AND (#4)  **Hits: 165** |

EMBASE

| **Search performed** |  |
| --- | --- |
| **Database** | EMBASE |
| **Account** |  |
| **Limits** | None |
| **Hits** |  |
| **Search** | ***Study design***  *Observation Study*  1#  'cohort study' OR 'studies, cohort' OR 'study, cohort' OR 'concurrent studies' OR 'studies, concurrent' OR 'concurrent study' OR 'study, concurrent' OR 'closed cohort studies' OR 'cohort studies, closed' OR 'closed cohort study' OR 'cohort study, closed' OR 'study, closed cohort' OR 'studies, closed cohort' OR 'cohort analysis' OR 'analyses, cohort' OR 'cohort analyses' OR 'historical cohort studies' OR 'cohort study, historical' OR 'historical cohort study' OR 'study, historical cohort' OR 'cohort studies, historical' OR 'studies, historical cohort' OR 'incidence studies' OR 'incidence study' OR 'studies, incidence' OR 'study, incidence' OR 'studies, case-control' OR 'study, case-control' OR 'case-comparison studies' OR 'case comparison studies' OR 'case-comparison study' OR 'studies, case-comparison' OR 'study, case-comparison' OR 'case-compeer studies' OR 'studies, case-compeer' OR 'case-referrent studies' OR 'case referrent studies' OR 'case-referrent study' OR 'studies, case-referrent' OR 'study, case-referrent' OR 'case-referent studies' OR 'case referent studies' OR 'case-referent study' OR 'studies, case-referent' OR 'study, case-referent' OR 'case-base studies' OR 'case base studies' OR 'studies, case-base' OR 'case control studies' OR 'case control study'  **Hits: 1.448.665**  *Low back pain*  2#  'back pain, low' OR 'back pains, low' OR 'low back pains' OR 'pain, low back' OR 'pains, low back' OR 'low back pain' OR 'lower back pain' OR 'back pain, lower' OR 'back pains, lower' OR 'lumbago' OR 'lower back pains' OR 'pain, lower back' OR 'pains, lower back' OR 'low back ache' OR 'ache, low back' OR 'aches, low back' OR 'back ache, low' OR 'back aches, low' OR 'low back aches' OR 'low backache' OR 'backache, low' OR 'backaches, low' OR 'low backaches' OR 'low back pain, postural' OR 'postural low back pain' OR 'low back pain, posterior compartment' OR 'low back pain, recurrent' OR 'recurrent low back pain' OR 'low back pain, mechanical' OR 'mechanical low back pain'  **Hits: 85.809**  ***HIP***  #3  hip OR hips OR 'hip joints' OR 'joint, hip' OR 'joints, hip' OR 'acetabulofemoral joint' OR 'acetabulofemoral joints' OR 'joint, acetabulofemoral' OR 'joints, acetabulofemoral'  **Hits: 293.782**  **Avaliação biomecânica**  #4  electromyographies OR electromyography OR 'surface electromyography' OR 'electromyographies, surface' OR 'electromyography, surface' OR 'surface electromyographies' OR electromyogram OR electromyograms OR 'dynamometer, muscle strength' OR 'dynamometers, muscle strength' OR 'muscle strength dynamometers' OR 'strength, muscle' OR 'arthrogenic muscle inhibition' OR 'arthrogenic muscle inhibitions' OR 'muscle strength' OR 'inhibition, arthrogenic muscle' OR biomechanics OR 'muscle inhibition, arthrogenic' OR 'phenomena, biomechanical' OR 'biomechanic phenomena' OR 'biomechanic phenomenas' OR 'phenomena, biomechanic' OR 'phenomenas, biomechanic' OR 'mechanobiological phenomena' OR 'phenomena, mechanobiological' OR kinematics OR 'performance test' OR 'endurance test' OR 'functional performance, physical' OR 'functional performances, physical' OR 'performance, physical functional' OR 'performances, physical functional' OR 'physical functional performances' OR 'functional performance' OR 'functional performances' OR 'performance, functional' OR 'performances, functional' OR 'physical performance' OR 'performance, physical' OR 'performances, physical' OR 'physical performances' OR 'muscle activation' OR 'development, muscle' OR 'muscular development' OR 'development, muscular' OR myogenesis OR myofibrillogenesis OR 'range of motion' OR 'passive range of motion' OR 'joint range of motion' OR 'joint mobility' OR 'flexibility, joint' OR 'joint flexibility'  **Hits: 574327**  **Final:97**  #5  #1 AND #2 AND #3 AND #4 |

CINAHL

| **Search performed** |  |
| --- | --- |
| **Database** | CINAHL |
| **Account** |  |
| **Limits** |  |
| **Hits** |  |
| **Search** | ***Study design***  *Observational study*  S1  ( ( ( ( "Cohort Study" OR "Studies, Cohort" OR "Study, Cohort" OR "Concurrent Studies" OR "Studies, Concurrent" OR "Concurrent Study" OR "Study, Concurrent" OR "Closed Cohort Studies" OR "Cohort Studies, Closed" OR "Closed Cohort Study" OR "Cohort Study, Closed" OR "Study, Closed Cohort" ) OR "Studies, Closed Cohort" OR "Analysis, Cohort" OR "Cohort Analysis" OR "Analyses, Cohort" OR "Cohort Analyses" OR "Historical Cohort Studies" OR "Cohort Study, Historical" OR "Historical Cohort Study" OR "Study, Historical Cohort" OR "Cohort Studies, Historical" OR "Studies, Historical Cohort" ) OR "Incidence Studies" OR "Incidence Study" OR "Studies, Incidence" OR "Study, Incidence" OR "Case-Control Study" OR "Studies, Case-Control" OR "Study, Case-Control" OR "Case-Comparison Studies" OR "Case Comparison Studies" OR "Case-Comparison Study" OR "Studies, Case-Comparison" ) OR "Study, Case-Comparison" OR "Case-Compeer Studies" OR "Studies, Case-Compeer" OR "Case-Referrent Studies" OR "Case Referrent Studies" OR "Case-Referrent Study" OR "Studies, Case-Referrent" OR "Study, Case-Referrent" OR "Case-Referent Studies" OR "Case Referent Studies" OR "Case-Referent Study" ) OR "Studies, Case-Referent" OR "Study, Case-Referent" OR "Case-Base Studies" OR "Case Base Studies" OR "Studies, Case-Base" OR "Case Control Studies" OR "Case Control Study"  **Hits: 238.916**  *Low back pain*  S2  ( ( "Low back pain" OR "Back Pain, Low" OR "Back Pains, Low" OR "Low Back Pains" OR "Pain, Low Back" OR "Pains, Low Back" OR Lumbago OR "Lower Back Pain" OR "Back Pain, Lower" OR "Back Pains, Lower" OR "Lower Back Pains" OR "Pain, Lower Back" ) OR "Pains, Lower Back" OR "Low Back Ache" OR "Ache, Low Back" OR "Aches, Low Back" OR "Back Ache, Low" OR "Back Aches, Low" OR "Low Back Aches" OR "Low Backache" OR "Backache, Low" OR "Backaches, Low" OR "Low Backaches" ) OR "Low Back Pain, Postural" OR "Postural Low Back Pain" OR "Low Back Pain, Posterior Compartment" OR "Low Back Pain, Recurrent" OR "Recurrent Low Back Pain" OR "Low Back Pain, Mechanical" OR "Mechanical Low Back Pain"  **Hits: 35.230**  ***Hip***  S3  Hip OR Hips OR "Hip Joints" OR "Joint, Hip" OR "Joints, Hip" OR "Acetabulofemoral Joint" OR "Acetabulofemoral Joints" OR "Joint, Acetabulofemoral" OR "Joints, Acetabulofemoral"  **Hits: 83.354**  **Avaliação biomecânica**  S4  ( ( ( ( Electromyographies OR Electromyography OR "Surface Electromyography" OR "Electromyographies, Surface" OR "Electromyography, Surface" OR surface electromyography OR "Surface Electromyographies" OR Electromyogram OR Electromyograms OR "Dynamometer, Muscle Strength" OR "Dynamometers, Muscle Strength" OR "Muscle Strength Dynamometers" ) OR "Strength, Muscle" OR "Arthrogenic Muscle Inhibition" OR "Arthrogenic Muscle Inhibitions" OR "Inhibition, Arthrogenic Muscle" OR Biomechanics OR "Muscle Inhibition, Arthrogenic" OR "Phenomena, Biomechanical" OR "Biomechanic Phenomena" OR "Biomechanic Phenomenas" OR "Phenomena, Biomechanic" OR "Phenomenas, Biomechanic" ) OR "Mechanobiological Phenomena" OR "Phenomena, Mechanobiological" OR Kinematics OR "Performance Test" OR "Endurance Test" OR "Functional Performance, Physical" OR "Functional Performances, Physical" OR "Performance, Physical Functional" OR "Performances, Physical Functional" OR "Physical Functional Performances" OR "Functional Performance" ) OR "Functional Performances" OR "Performance, Functional" OR "Performances, Functional" OR "Physical Performance" OR "Performance, Physical" OR "Performances, Physical" OR "Physical Performances" OR "muscle activation" OR "Development, Muscle" OR "Muscular Development" OR "Development, Muscular" ) OR Myogenesis OR Myofibrillogenesis OR "Range of Motion" OR "Joint Range of Motion" OR "Joint Flexibility" OR "Flexibility, Joint" OR "Range of Motion" OR "Passive Range of Motion" OR "Joint Range of Motion" OR "Joint Flexibility" OR "Flexibility, Joint"  **Hits: 124.344**  ***Final connections***  S5 S1 AND S2 AND S3 AND S4  **Hits: 3** |
|  |  |

SPORTDiscus

| **Search performed** |  |
| --- | --- |
| **Database** | SPORTDiscus |
| **Limits** | None |
| **Hits** |  |
| **Search** | ***Study design***  *Observational sutdy*  #1  ((((((((((((((((((((((((((((((((((((((((((((((((((("Cohort Study") OR ("Studies, Cohort")) OR ("Study, Cohort")) OR ("Concurrent Studies")) OR ("Studies, Concurrent")) OR ("Concurrent Study")) OR ("Study, Concurrent")) OR ("Closed Cohort Studies")) OR ("Cohort Studies, Closed")) OR ("Closed Cohort Study")) OR ("Cohort Study, Closed")) OR ("Study, Closed Cohort")) OR ("Studies, Closed Cohort")) OR ("Analysis, Cohort")) OR ("Cohort Analysis")) OR ("Analyses, Cohort")) OR ("Cohort Analyses")) OR ("Historical Cohort Studies")) OR ("Cohort Study, Historical")) OR ("Historical Cohort Study")) OR ("Study, Historical Cohort")) OR ("Cohort Studies, Historical")) OR ("Studies, Historical Cohort")) OR ("Incidence Studies")) OR ("Incidence Study")) OR ("Studies, Incidence")) OR ("Study, Incidence")) OR ("Case-Control Study")) OR ("Studies, Case-Control")) OR ("Study, Case-Control")) OR ("Case-Comparison Studies")) OR ("Case Comparison Studies")) OR ("Case-Comparison Study")) OR ("Studies, Case-Comparison")) OR ("Study, Case-Comparison")) OR ("Case-Compeer Studies")) OR ("Studies, Case-Compeer")) OR ("Case-Referrent Studies")) OR ("Case Referrent Studies")) OR ("Case-Referrent Study")) OR ("Studies, Case-Referrent")) OR ("Study, Case-Referrent")) OR ("Case-Referent Studies")) OR ("Case Referent Studies")) OR ("Case-Referent Study")) OR ("Studies, Case-Referent")) OR ("Study, Case-Referent")) OR ("Case-Base Studies")) OR ("Case Base Studies")) OR ("Studies, Case-Base")) OR ("Case Control Studies")) OR ("Case Control Study")  **Hits: 16.272**  *Low back pain*  #2  ((((((((((((((((((((((((((((("Low back pain") OR ("Back Pain, Low")) OR ("Back Pains, Low")) OR ("Low Back Pains")) OR ("Pain, Low Back")) OR ("Pains, Low Back")) OR (Lumbago)) OR ("Lower Back Pain")) OR ("Back Pain, Lower")) OR ("Back Pains, Lower")) OR ("Lower Back Pains")) OR ("Pain, Lower Back")) OR ("Pains, Lower Back")) OR ("Low Back Ache")) OR ("Ache, Low Back")) OR ("Aches, Low Back")) OR ("Back Ache, Low")) OR ("Back Aches, Low")) OR ("Low Back Aches")) OR ("Low Backache")) OR ("Backache, Low")) OR ("Backaches, Low")) OR ("Low Backaches")) OR ("Low Back Pain, Postural")) OR ("Postural Low Back Pain")) OR ("Low Back Pain, Posterior Compartment")) OR ("Low Back Pain, Recurrent")) OR ("Recurrent Low Back Pain")) OR ("Low Back Pain, Mechanical")) OR ("Mechanical Low Back Pain")  **Hits: 8.195**  ***Hip***  #3  ((((((((Hip) OR (Hips)) OR ("Hip Joints")) OR ("Joint, Hip")) OR ("Joints, Hip")) OR ("Acetabulofemoral Joint")) OR ("Acetabulofemoral Joints")) OR ("Joint, Acetabulofemoral")) OR ("Joints, Acetabulofemoral")  **Hits: 31.928**  **Avaliação biomecânica**  #4  (((((((((((((((((((((((((((((((((((((((((((((((((((((((((Electromyographies) OR (Electromyography)) OR ("Surface Electromyography")) OR ("Electromyographies, Surface")) OR ("Electromyography, Surface")) OR ("Surface Electromyographies")) OR (Electromyogram)) OR (Electromyograms)) OR ("Dynamometer, Muscle Strength")) OR ("Dynamometers, Muscle Strength")) OR ("Muscle Strength Dynamometers")) OR ("Strength, Muscle")) OR ("Arthrogenic Muscle Inhibition")) OR ("Arthrogenic Muscle Inhibitions")) OR ("Inhibition, Arthrogenic Muscle")) OR (Biomechanics)) OR ("Muscle Inhibition, Arthrogenic")) OR ("Phenomena, Biomechanical")) OR ("Biomechanic Phenomena")) OR ("Biomechanic Phenomenas")) OR ("Phenomena, Biomechanic")) OR ("Phenomenas, Biomechanic")) OR ("Mechanobiological Phenomena")) OR ("Phenomena, Mechanobiological")) OR (Kinematics)) OR ("Performance Test")) OR ("Endurance Test")) OR ("Functional Performance, Physical")) OR ("Functional Performances, Physical")) OR ("Performance, Physical Functional")) OR ("Performances, Physical Functional")) OR ("Physical Functional Performances")) OR ("Functional Performance")) OR ("Functional Performances")) OR ("Performance, Functional")) OR ("Performances, Functional")) OR ("Physical Performance")) OR ("Performance, Physical")) OR ("Performances, Physical")) OR ("Physical Performances")) OR ("muscle activation")) OR ("Development, Muscle")) OR ("Muscular Development")) OR ("Development, Muscular")) OR (Myogenesis)) OR (Myofibrillogenesis)) OR ("Range of Motion")) OR ("Joint Range of Motion")) OR ("Joint Flexibility")) OR ("Flexibility, Joint")) OR ("Range of Motion")) OR ("Passive Range of Motion")) OR (“Joint motion”)) OR ("Joint Range of Motion")) OR ("Joint Flexibility")) OR ("Flexibility, Joint")) OR ("Range of Motion")) OR ("Passive Range of Motion")  **Hits: 112.566**  ***Final connections***  S5 S1 AND S2 AND S3 AND S4  **Hits: 1** |
